# Accounting for parameter uncertainty in the definition of parametric distributions used to describe individual patient variation in health economic models

**DOI:** 10.1186/s12874-017-0437-y

**Published:** 2017-12-15

**Authors:** Koen Degeling, Maarten J. IJzerman, Miriam Koopman, Hendrik Koffijberg

**Affiliations:** 1 Health Technology and Services Research Department, MIRA institute for Biomedical Technology and Technical Medicine, University of Twente, P.O. Box 217, 7500 AE Enschede, The Netherlands; 2 Department of Medical Oncology, University Medical Centre, Huispost B02.225, P.O. Box 85500, 3508 GA Utrecht, The Netherlands

**Keywords:** Patient-level variation, Stochastic uncertainty, Parameter uncertainty, State-transition modeling, Discrete event simulation, Personalized medicine, Individual patient data

## Abstract

**Background:**

Parametric distributions based on individual patient data can be used to represent both stochastic and parameter uncertainty. Although general guidance is available on how parameter uncertainty should be accounted for in probabilistic sensitivity analysis, there is no comprehensive guidance on reflecting parameter uncertainty in the (correlated) parameters of distributions used to represent stochastic uncertainty in patient-level models. This study aims to provide this guidance by proposing appropriate methods and illustrating the impact of this uncertainty on modeling outcomes.

**Methods:**

Two approaches, 1) using non-parametric bootstrapping and 2) using multivariate Normal distributions, were applied in a simulation and case study. The approaches were compared based on point-estimates and distributions of time-to-event and health economic outcomes. To assess sample size impact on the uncertainty in these outcomes, sample size was varied in the simulation study and subgroup analyses were performed for the case-study.

**Results:**

Accounting for parameter uncertainty in distributions that reflect stochastic uncertainty substantially increased the uncertainty surrounding health economic outcomes, illustrated by larger confidence ellipses surrounding the cost-effectiveness point-estimates and different cost-effectiveness acceptability curves. Although both approaches performed similar for larger sample sizes (i.e. *n* = 500), the second approach was more sensitive to extreme values for small sample sizes (i.e. *n* = 25), yielding infeasible modeling outcomes.

**Conclusions:**

Modelers should be aware that parameter uncertainty in distributions used to describe stochastic uncertainty needs to be reflected in probabilistic sensitivity analysis, as it could substantially impact the total amount of uncertainty surrounding health economic outcomes. If feasible, the bootstrap approach is recommended to account for this uncertainty.

**Electronic supplementary material:**

The online version of this article (doi: 10.1186/s12874-017-0437-y) contains supplementary material, which is available to authorized users.

## Background

Clinical decision-making is aiming towards patient-specific and preference-sensitive treatment, based on multiple biomarkers for treatment targeting and monitoring patients’ response to treatment [1]. Consequently, there is an increasing need for corresponding patient-level models to accurately represent clinical practice when estimating the health economic impact of novel healthcare interventions [2, 3]. To facilitate decision making, such models should adequately reflect all types of uncertainty in the synthesized evidence used for analysis [4]. This is particularly relevant in patient-level modeling studies in which reflecting patient heterogeneity may effectively increase uncertainty, for example by relatively low sample sizes in defined subgroups or by an increasing number of parameters that need to be estimated to account for patient characteristics in individualized predictions.

Uncertainty in evidence can be disaggregated into stochastic uncertainty (i.e. patient-level variation or first-order uncertainty) and parameter uncertainty (i.e. second-order uncertainty) [4]. This can be illustrated using a time-to-event parameter, e.g. the time-to-progression after surgery. If, for a certain patient group, this parameter is defined by a mean estimate (Table 1 – Box A), the parameter uncertainty in this mean estimate needs to be accounted for, which can be done using a parametric distribution in the probabilistic sensitivity analysis (PSA) (Table 1 – Box B) [4]. For example, this parameter uncertainty could be reflected by defining a Normal distribution for the mean time-to-progression based on the estimated mean and standard error, by applying the Central Limit Theorem [5]. Additionally, it is possible to account for stochastic uncertainty by using parametric distributions to describe individual patient variation, such as typically used in patient-level state-transition models [6] and discrete event simulation (DES) models [7]. For example, a Weibull distribution [8] can be used in a DES model to derive and assign patient-specific time-to-progression values (Table 1 – Box C), rather than assigning the estimated mean time-to-progression to all patients (Table 1 – Box A). As for the parameter defined by the mean estimate in Box A of Table 1, the uncertainty in the parameters defining this Weibull distributions needs to be accounted for [4]. However, there is currently no clear guidance on *how to reflect parameter uncertainty* (i.e. second-order uncertainty) in these *parametric distributions used to describe stochastic uncertainty* (i.e. patient-level variation or fist-order uncertainty) in PSA (Table 1 – Box D).

**Table 1:**
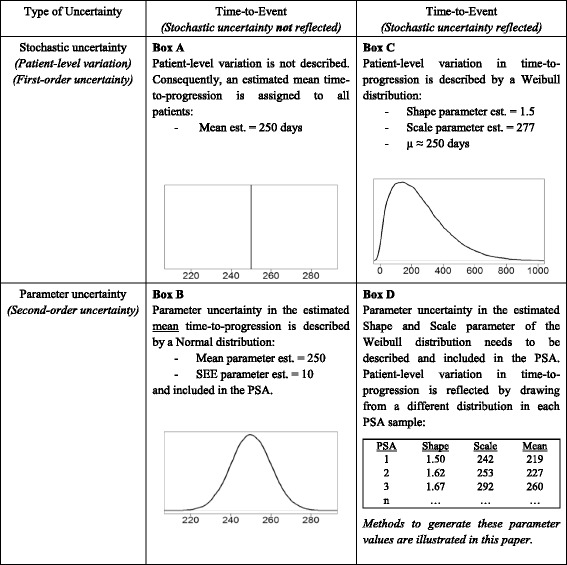
Illustration of the example that is discussed in the introduction

*SEE* standard error of the estimate

Reflecting parameter uncertainty in parametric distributions is challenging because these distributions are typically defined by multiple parameters, e.g. the shape and scale parameter of the Weibull distribution, with their values preferably estimated from individual patient data (IPD). When estimated from IPD, these distributions’ parameters are correlated for most of the parametric distributions commonly used in health economic analyses, such as Gamma, Beta, Log-Normal, and Weibull distributions [8]. Consequently, it is incorrect to estimate the values of, for example, the shape and scale parameters independently, and to define independent (i.e. separate) distributions for each of these parameters’ uncertainty in PSA. Therefore, guidance is needed towards approaches that maintain the correlation in the parameters of distributions reflecting stochastic uncertainty when accounting for the parameter uncertainty these parameters introduce.

The lack of specific guidance is illustrated by recently published modeling studies that do not fully utilize IPD while performing PSA, for example, by adjusting predicted values (stochastic uncertainty) with a random percentage [9], by excluding correlation between parametric distributions’ parameters [10], or altogether not reporting (including) parameter uncertainty in parametric distributions [11]. As a consequence of inadequately reflecting all types of uncertainty in the synthesized evidence used for analysis, suboptimal resource allocation and research prioritization decisions may be made due to biased outcomes of PSA, overestimated confidence in the corresponding expected values of the PSA, and ensuing biased estimation of the value of collecting additional evidence to better inform decision making [12].

The objective of this study is to provide explicit guidance for health economic modelers on how parameter uncertainty in parametric distributions used to describe stochastic uncertainty (i.e. patient-level variation) can be considered in PSA. In order to do so, two alternative solutions are illustrated and compared: 1) a non-parametric bootstrapping approach and 2) an approach using multivariate Normal distributions. Both approaches are used in a DES simulation study and in a DES case study in which stochastic uncertainty in time-to-event data is described by parametric distributions. Additionally, the potential impact of increased parameter uncertainty due to subgroup stratification on health economic outcomes is illustrated.

## Methods

**Fig. 1:**
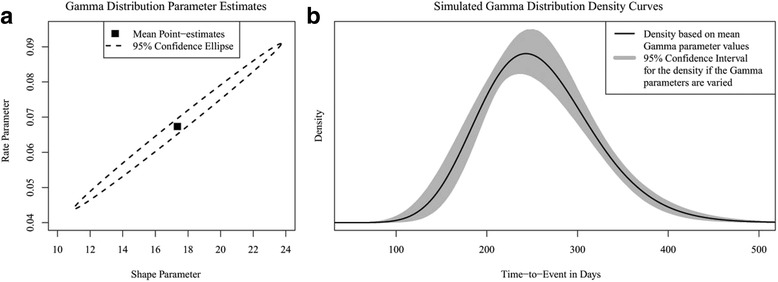
**a** Exemplary plot of a Gamma distribution’s shape and scale parameters, showing their point-estimates and the 95% confidence ellipse representing the uncertainty and correlation in these estimates. **b** Density plot of the Gamma distribution as defined in Fig. 1a. The black line represents the density curve from which individual values are drawn in each run of the PSA if parameter uncertainty in the parameter estimates is ignored. The grey surface represents the 95% confidence interval of density curves from which individual values are drawn if different (correlated) sets of parameter values are used in each run of the PSA, i.e. if parameter uncertainty in the parameter estimates is accounted for

Consider the scenario in which time-to-event observations from a clinical study are used to describe stochastic uncertainty by fitting a Gamma distribution [8], i.e. the distributions’ (correlated) shape and rate parameter values are estimated from the IPD. Figure 1a shows an example of the estimated values of these parameters, including the 95% confidence ellipse, representing the sets (i.e. combinations) of parameter values. If uncertainty in the parameter estimates (i.e. parameter uncertainty) is ignored by using the parameters’ point-estimates in each run of the PSA, individual time-to-event values would be repeatedly drawn based on the same mean density curve of Fig. 1b in all runs of the PSA. However, if this parameter uncertainty is reflected by using different sets of correlated parameter estimates for each run of the PSA, a variety of distributions is simulated, which is illustrated by the 95% confidence interval (surface) in Fig. 1b. As this variety of distributions reflects uncertainty in the individual time-to-event values as well as overall (mean) time-to-event values, this uncertainty can be seen as parameter uncertainty (in this case of the ‘hyperparameters’, i.e. the parameters defining the time-to-event distribution). Both proposed approaches, which are introduced subsequently, can be used to generate these correlated sets of distributions’ parameter values based on IPD. A different set of generated parameters values can be used in the PSA, incorporating one set of correlated values in each Monte Carlo sample.

### Non-parametric bootstrapping (Bootstrap approach)

Non-parametric bootstrapping is a statistical technique that can be used to construct an approximate sampling distribution of a statistic of interest, without the need for assumptions regarding the distribution of this statistic [13]. It has been applied in health economics, for example, to construct confidence intervals for the incremental net benefit in economic evaluations alongside clinical trials [5, 14]. In the Bootstrap approach, the distributions’ parameters are repeatedly estimated based on different bootstrap samples of the original dataset, which are obtained by resampling the original dataset with replacement, such that the size of the bootstrap sample equals the size of the original dataset [13]. A detailed discussion of the Bootstrap approach is provided in Additional file 1. Briefly, reflecting parameter uncertainty with this approach consists of the following four steps:

1. Generate a feasible* bootstrap sample of the original dataset, by resampling this dataset with replacement, such that the sample size of the bootstrap sample equals that of the original dataset.
2. Fit the pre-specified distribution(s)** to the bootstrap sample and record the estimated parameter values.
3. Repeat (1) and (2) ***r*** times, where ***r*** equals the required number of PSA runs.
4. Perform the PSA, using a different set of parameter values to define the distribution(s) for each PSA run.

* The definition of feasible bootstrap samples may vary between studies. Please see Additional file 1 for a more in-depth discussion.

** Note that if multiple distributions are fitted in step (2), all distributions need to be fitted on the same bootstrap sample to preserve correlation among all distributions and other parameters used to describe variables in the dataset.

### Multivariate normal distributions (MVNorm approach)

The MVNorm approach assumes the distributions’ parameters to be Normal distributed, which is valid for sufficiently large sample sizes according to the Central Limit Theorem [5], as suggested by Briggs et al. for regression models in general [15]. Given a distribution’s parameter estimates and their variance-covariance matrix, a multivariate Normal distribution can be defined and used to draw correlated sets of parameter values. A detailed discussion of the MVNorm approach is provided in Additional file 1. Briefly, reflecting parameter uncertainty with this approach consists of the following four steps:

1. Fit the pre-specified distribution to the original dataset and record the estimated parameter values and (calculate) the variance-covariance matrix.
2. Define a multivariate Normal distribution from the parameters’ estimates and their variance-covariance matrix according to (1).
3. Draw ***r*** feasible* sets of parameter values from the defined distribution (2), where ***r*** equals the required number of PSA runs.
4. Perform the PSA, using a different set of parameter values to define the distribution(s) for each PSA run.
  * The drawn sets of parameter values need to be assessed for their feasibility, i.e. whether the parameter values are appropriate for the pre-specified distributions. Please see Additional file 1 for a more in-depth discussion.

### Simulation study

**Fig. 2:**
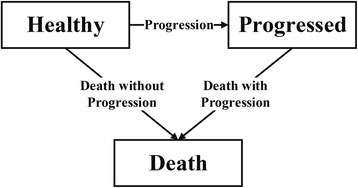
Graphical representation of the DES model that was used for the simulation study as described in the Methods section

A simulation study was performed to assess potential differences in the performance of both approaches and compare them to the scenario in which parameter uncertainty in the time-to-event distributions was not considered. This simulation study was performed in R Statistical Software version 3.3.2 [16] and used a basic health economic DES model to compare two treatment strategies in terms of health economic outcomes. This health economic DES model was structured according to a basic three state disease progression model, i.e. healthy, progressed, and death, and included two competing risks for patients in the healthy state, i.e. progression and death (Fig. 2). Time-to-event data was simulated using Weibull distributions, separately for the intervention and control patient populations, which differed in terms of survival and treatment costs in the progressed state. The exact definitions of the time-to-event distributions and cost and effect parameters are provided in Additional file 1.

**Fig. 3:**
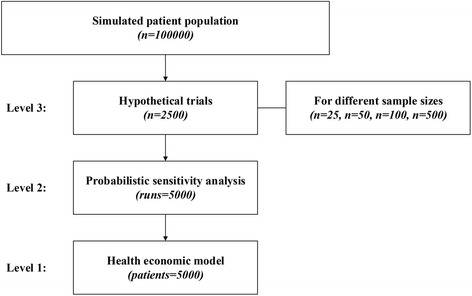
Graphical representation of the simulation study as described in the Methods section

The simulation study was carried out for several sample sizes, i.e. 500, 100, 50, and 25 patients, performing 2500 simulation runs (i.e. hypothetical trials) of 5000 PSA runs, each including 5000 patients per treatment strategy, for each sample size (Fig. 3). Weibull distributions were used to describe stochastic uncertainty in time-to-event data to avoid potential bias due to mismatching distributions, as Weibull distributions were also used to simulate the hypothetical populations. Distributions were fitted using the *fitdist* function of the *fitdistrplus* package [17] in R Statistical Software [16]. Stochastic uncertainty in the model’s cost and effect parameters was deliberately not considered to represent the common scenario in which IPD is not available for all model parameters, and PSA samples for these parameters were generated according to the distributions as defined in Additional file 1. Random draws from a multivariate Normal distribution were performed using the *mvrnorm* function of the *MASS* package [18].

Estimations of the “true” mean value and distribution of the parameters and health economic outcomes were obtained by analyzing the model for 2500 different samples from the simulated populations, with corresponding sample sizes. This resembles the scenario in which the population values, i.e. values based on observing the total population, would be approximated in practice by performing 2500 clinical studies. These “true” results were used to compare the generated sets of distributions’ parameter values and the health and economic outcomes using either of both approaches. The distributions of the time-to-event distributions’ parameter values and health economic outcomes were compared based on their relative entropy, i.e. the Kullback-Leibler divergence [19], using the *KLdiv* function of the *flexmix* package [20–22]. The relative entropy is a measure of the difference between two probability distributions, for which lower values indicate a better match of distributions. Additionally, the impact of considering the parameter uncertainty in the time-to-event distributions’ parameters was illustrated in an incremental cost-effectiveness plane for one hypothetical trial, i.e. one random run out of 2500 simulation runs, and in mean cost-effectiveness acceptability curves (CEACs), including a 95% confidence interval, based on all simulation runs.

### Case study

To illustrate how parameter uncertainty in time-to-event distributions’ parameter estimates could impact health economic outcomes in practice, a case study was performed based on the randomized phase 3 CAIRO3 study (NCT00442637) that was carried out by the Dutch Colorectal Cancer Group [23]. A total of 558 metastatic colorectal patients with stable disease or better after six cycles of capecitabine, oxaliplatin, and bevacizumab (CAPOX-B) induction therapy were randomized to either receive capecitabine and bevacizumab maintenance treatment or observation until progression of disease. CAPOX-B treatment was to be re-introduced upon progression on either maintenance or observation, and continued until second progression (PFS2) the primary endpoint of the study.

**Fig. 4:**
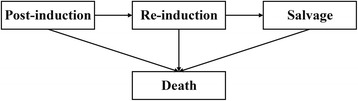
Graphical representation of the DES model that was used for the case study as described in the Methods section

A previous developed DES model was adapted to use the sets of distributions’ parameter values generated by the Bootstrap and MVNorm approach in the PSA (Additional file 1). The model was developed and validated in AnyLogic multimethod simulation software [24] according to good research practices guidelines [7, 25, 26], and structured according to the same health states as the state-transition model used for the original evaluation of the CAIRO3 study: post-induction, re-induction, salvage, and death [27] (Fig. 4). Event-specific probabilities and Weibull distributions were used to describe time-to-event data and handle the competing risks of disease progression and death in the post-induction and re-induction state [28]. Parameters of the distributions used to reflect parameter uncertainty in non-time-to-event parameters, e.g. costs and utilities, were deliberately defined exactly as in the original evaluation of the CAIRO3 study for all subgroups [27], so that observed differences in outcomes could be designated specifically to the uncertainty in time-to-event distributions’ parameter estimates. The health economic outcomes for all case study analyses were presented in incremental cost-effectiveness planes and CEACs based on 10,000 PSA runs of 10,000 patients per treatment strategy.

Clinical relevant subgroups were defined to resemble the personalized context described in the introduction, illustrating the impact of parameter uncertainty in time-to-event distributions’ parameter estimates on health economic outcomes for different sample sizes. Patients were stratified according to their treatment response (stable disease (SD) versus complete or partial response (CR/PR)) and stage of disease (synchronous versus metachronous), which resulted in a total of 8 subgroups with sample sizes ranging from 50 to 410 (Table 2). As illustrated in Table 2, this stratification created subgroups in which events were observed only once, or not all, which prohibits fitting of a Weibull distribution, and the probability of that event occurring was therefore set to zero.

**Table 2:** Overall and treatment strategy-specific subgroup definitions, sample sizes, and number of events for the case study

	Sample size	Event observations				
		Progression (Post-induction)	Death (Post-induction)	Progression (Re-induction)	Death (Re-induction)	Death (Salvage)
Subgroup 0: No Subgroups	558	519	39	370	109	410
Observation	279	270	9	200	54	216
Maintenance	279	249	30	170	55	194
Subgroup 1: SD	191	185	6	128	43	142
Observation	95	95	0	68	22	73
Maintenance	96	90	6	60	21	69
Subgroup 2: CR/PR	367	334	33	242	66	268
Observation	184	175	9	132	32	143
Maintenance	183	159	24	110	34	125
Subgroup 3: Synchronous	410	382	28	275	82	300
Observation	191	186	5	143	33	153
Maintenance	219	196	23	132	49	147
Subgroup 4: Metachronous	147	137	10	95	27	110
Observation	88	84	4	57	21	63
Maintenance	59	53	6	38	6	47
Subgroup 5: SD & Synchronous	141	136	5	94	34	102
Observation	67	67	0	49	15	52
Maintenance	74	69	5	45	19	50
Subgroup 6: SD & Metachronous	50	49	1	34	9	40
Observation	28	28	0	19	7	21
Maintenance	22	21	1	15	2	19
Subgroup 7: CR/PR & Synchronous	269	246	23	181	48	198
Observation	124	119	5	94	18	101
Maintenance	145	127	18	87	30	97
Subgroup 8: CR/PR & Metachronous	97	88	9	61	18	70
Observation	60	56	4	38	14	42
Maintenance	37	32	5	23	4	28

*SD* stable disease, *CR/PR* complete or partial response

## Results

### Simulation study

**Fig. 5:**
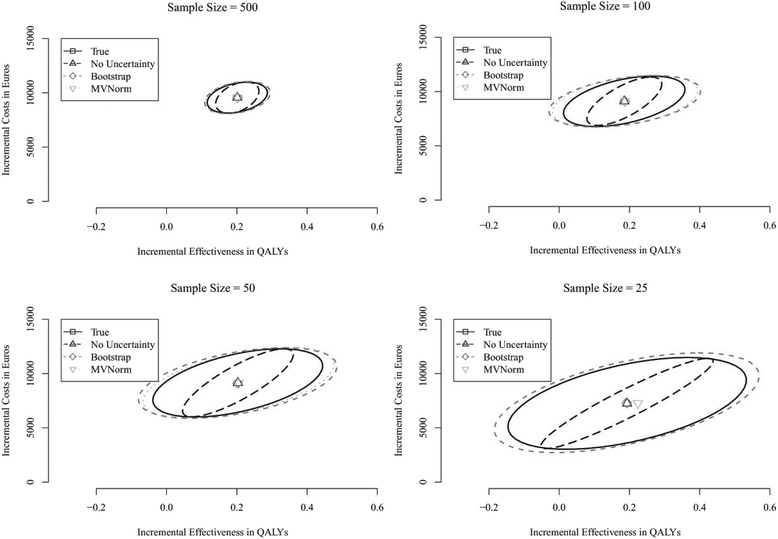
Incremental cost-effectiveness plane for one run of the simulation study showing the mean point-estimates and corresponding 95% confidence ellipses for the different sample sizes. The confidence ellipse for the MVNorm approach with sample size *n* = 25 was not plotted, because it was unrealistic large

The potential impact of considering parameter uncertainty in time-to-event distributions’ parameter estimates in PSA is illustrated for several sample sizes in Fig. 5, which shows results of one single run of the simulation study. Ignoring parameter uncertainty in the time-to-event distributions’ parameter estimates leads to an underestimation of the uncertainty surrounding cost-effectiveness outcomes. This is illustrated by the smaller confidence ellipse for this scenario (long-dashed black ellipse) compared to other scenarios in which uncertainty in the time-to-event distributions’ parameter estimates is accounted for using one of the proposed approaches (dashed gray and dotted light-gray ellipses). The indicated effect is already observed for rather large sample sizes (i.e. *n* = 500) and increases as sample size decreases, illustrated by the larger distance between the long-dashed black confidence ellipse and the dashed gray and dotted light-gray confidence ellipses for smaller sample sizes. Although the Bootstrap (dashed gray ellipse) and MVNorm approach (dotted light-gray ellipse) yield incremental cost-effectiveness point-estimates similar to the “real” value, both approaches slightly overestimate the magnitude of the uncertainty for sample sizes of *n* = 100 and smaller, demonstrated by smaller confidence ellipses for the “real” uncertainty (solid black ellipse). For very small sample sizes (i.e. *n* = 25), the MVNorm approach generates unrealistic parameter values, e.g. indicating a mean survival far beyond life-expectancy, leading to extreme and unrealistic health economic outcomes, which results in an unrealistic large confidence ellipse (not presented). However, since Fig. 5 represents only one run of the simulation study, these results may not be representative for the approaches in general.

**Fig. 6:**
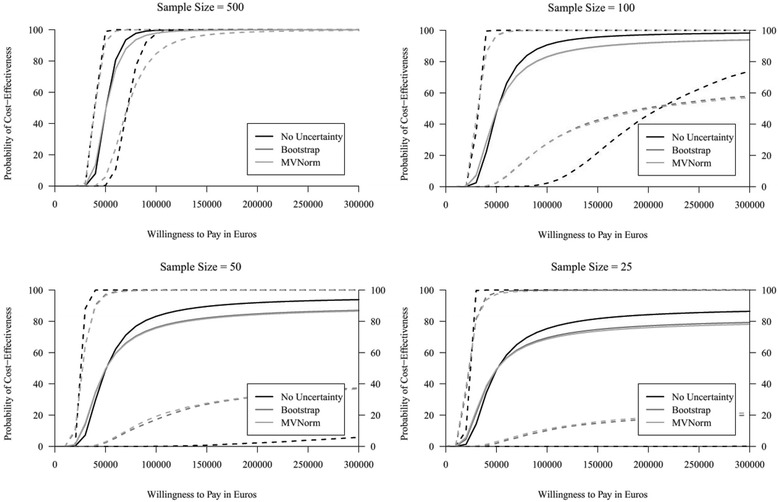
CEACs showing the mean probability of cost-effectiveness (solid lines) and corresponding 95% confidence interval (dashed lines) for all runs of the simulation study

To assess the performance of the approaches in general, 2500 of these comparisons were performed in the simulation study. Results show that both approaches yield comparable mean parameter estimates and standard errors (Additional file 1). Although the MVNorm approach seems to perform slightly better for small sample sizes (*n* = 25) in terms of mean parameter estimates, this approach too often yields extreme and unrealistic outcomes in the health economic simulation. Considering the Kullback-Leibler divergence, both approaches perform similar, though on average the MVNorm approach seems to represent the “estimated true” distributions slightly better for very small sample sizes (i.e. *n* = 25) (Additional file 1). Also the CEACs presented in Fig. 6 show that the results for both approaches are similar (light gray and gray lines). However, compared to the results of the strategy in which the uncertainty in time-to-event distributions’ parameter estimates is not considered (black line), both approaches yield different mean CEACs (solid lines) with different confidence intervals (dashed lines), illustrating the potential health economic impact of ignoring this uncertainty.

### Case study

**Fig. 7:**
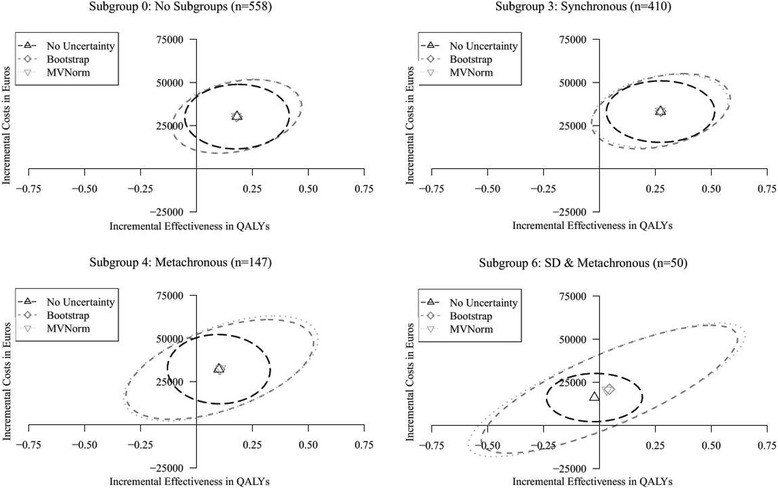
Incremental cost-effectiveness planes for the cohort (Subgroup 0) and Subgroups 3, 4, and 6 of the case study, showing the mean point-estimates and corresponding 95% confidence ellipses

Incremental cost-effectiveness planes for the cohort analysis and selected subgroup analyses of the case study are presented in Fig. 7. CEACs and incremental cost-effectiveness planes for the cohort analysis and all subgroups analyses are available in Additional file 1. The cost-effectiveness point-estimate for the cohort (Subgroup 0) is not affected by considering the parameter uncertainty in the time-to-event distributions’ parameter estimates in PSA, as the points in the corresponding incremental cost-effectiveness plane overlap. However, there is a substantial increase in the amount of uncertainty surrounding this point-estimate when the uncertainty in the distributions’ parameter estimates is accounted for, illustrated by the distance between the long-dashed black confidence ellipse and the dashed gray and dotted light-gray confidence ellipses. The potential impact of this increase in uncertainty is illustrated by the results for Subgroup 3. Without considering the parameter uncertainty in the distributions’ parameter estimates, the results indicate that health loss due to maintenance treatment (experimental strategy) is unlikely in this subgroup, as the corresponding (dashed black) confidence ellipse is entirely located right of the vertical axis. However, the dashed gray and dotted light-gray confidence ellipses, including this uncertainty, show there actually is a non-zero probability of health loss, as these are partly located left of the vertical axis.

As the cohort is stratified, and sample size decreases, the impact of considering parameter uncertainty in the time-to-event distributions’ parameter estimates in PSA increases substantially, which is illustrated by the results for Subgroup 4 and Subgroup 6. These results show that the increase in uncertainty due to stratification may become so large that (further) subgroup stratification might not be informative. Additionally, the results for Subgroup 6 illustrate that for small sample sizes (i.e. *n* = 50) the point-estimates of the cost-effectiveness outcomes themselves may also be affected by the uncertainty in the distributions’ parameters, which is illustrated by the non-overlapping points in the corresponding incremental cost-effectiveness plot.

No meaningful differences in the point estimates and the magnitude of uncertainty surrounding these estimates between the Bootstrap and MVNorm approach are observed, as their point-estimates and confidence ellipse overlap to a great extent. However, as also observed in the simulation study, the use of the MVNorm approach occasionally results in extreme and unlikely parameter values, which were to be excluded from the simulation.

## Discussion

As demonstrated in this paper, parameter uncertainty in parametric distributions used to describe stochastic uncertainty (i.e. patient-level variation) should be explicitly accounted for in PSA by modelers, as it does impact incremental cost-effectiveness point-estimates and CEACs. If this type of uncertainty is ignored, suboptimal resource allocations or research prioritization decisions may be made, due to an underestimation of the total uncertainty surrounding health economic outcomes. This is particularly relevant in a personalized treatment context in which patient stratification obviously leads to an increase in uncertainty on the level of subgroups compared to the level of the full patient group due to decreasing sample sizes in subgroups. This increase in uncertainty should of course be reflected in PSA, and although it is likely that many experienced modelers already do so, clear guidance on appropriate methods was not yet available. Besides time-to-event distributions, this notion also applies to other types of distributions that are used to reflect stochastic uncertainty, e.g. Gamma distributions to describe patient-level costs. Although DES was used in both the simulation and case study, these findings apply to any patient-level modeling method used to reflect stochastic uncertainty, e.g. microsimulation state-transition models. Reflecting all parametric uncertainty in this way does require drawing values from a different distribution in each PSA sample, providing some additional work for modelers. However, the required effort is minimal and an online tool, including tutorials, has been made available alongside this paper to easily analyze individual patient data for implementation into patient-level models [29].

When accounting for parameter uncertainty in distributions’ parameter estimates, the Bootstrap approach has some advantages over the MVNorm approach. The Bootstrap approach seems more robust for smaller sample sizes, does not require any assumptions regarding underlying distributions, and preserves the correlation in the whole dataset. Although not distinctively illustrated in this study, the latter also concerns the correlation with other, non-time-to-event, related parameters, such as utilities and costs, and can be considered a major advantage. The Bootstrap approach does, however, require the definition of a feasible bootstrap sample. Especially in case of scarce events, which are inevitable in personalized medicine, this can be challenging. Moreover, the sets of parameters values probably need to be generated outside the software environment used for the simulation and need to be imported into this environment for performing the PSA.

Although the MVNorm approach is better capable of handling scarce events in theory, mainly because it does not require the definition of a feasible bootstrap sample, it has several severe downsides. The MVNorm approach requires the definition of feasible values for the distributions’ parameter estimates and the assumption that these estimates follow a Normal distribution, which is not appropriate for insufficiently large sample sizes. For example, the shape and rate parameter of the Gamma distribution may be skewed, depending on the IPD on which their values are estimated. Additionally, many of the commonly used parametric distributions are defined for positive parameter values only, whereas Normal distributions are defined for any real number, including negatives. Furthermore, although this approach is better capable of handling scarce events in theory, it is likely to yield extreme parameter values for smaller sample sizes due to increasing standard errors, a scenario in which the use of the MVNorm approach is advised against.

Given the advantages and disadvantages of both approaches, the Bootstrap approach is recommended over the MVNorm approach if constructing enough feasible bootstrap samples is possible. However, if modelers feel the need to use the MVNorm approach, for example because multivariate Normal distributions are supported in the used software environment and the Bootstrap approach is not, they should carefully check whether 1) the sample size is sufficient, i.e. no implausible and extreme parameter values are observed, and 2) whether correlation between defined distributions to reflect patient-level variation is low or negligible and the additional uncertainty introduced by using independent multivariate Normal distributions (e.g. for costs, utilities, time-to-events) is therefore limited.

Several choices in the study design may have influenced the final results. Both the simulation study and case study focus on the impact of parameter uncertainty specifically in time-to-event distributions, because primary outcomes in clinical studies are often related to time-to-events, e.g. overall survival, and these distributions characterize DES. Additionally, accounting for the parameter uncertainty in non-time-to-event related distributions may further increase the amount of uncertainty surrounding the health economic outcomes, which will contribute to the conclusion that parameter uncertainty in distributions used to describe stochastic uncertainty (i.e. patient-level variation) needs to be accounted for. Furthermore, Weibull distributions are used to describe the IPD, which is a design choice and therefore introduces structural uncertainty. This choice is not expected to meaningfully influence the outcomes, because the fitted distributions match the data well. Moreover, other distributions’ parameters may have a stronger correlation, which would further stress the need to use one of the proposed approaches to appropriately account for the uncertainty in these parameters.

Further research may be directed towards evaluating the impact of using different types of distributions to describe stochastic uncertainty on health economic outcomes, the uncertainty surrounding these outcomes, and the performance of both approaches. Additionally, sensitivity analyses other than PSA, such as deterministic sensitivity analysis and structural sensitivity analysis, might be considered. Furthermore, additional guidance is desirable on how uncertainty in IPD can be appropriately combined with uncertainty in aggregated data on population level, e.g. a reported mean estimate and standard error from literature, for sensitivity analyses in patient-level models.

## Conclusions

With an increasing need for patient-level models to accurately represent clinical practice, modelers should be aware that the parameter uncertainty in parametric distributions used to describe stochastic uncertainty (i.e. patient-level variation) should be accounted for in PSA performed in health economic modeling studies. This type of uncertainty could have a substantial impact on the total amount of uncertainty surrounding the health economic outcomes and may influence healthcare decision-making. At least two approaches are available to account for the parameter uncertainty in parametric distributions used to describe stochastic uncertainty. If feasible, the Bootstrap approach is recommended to account for this type of uncertainty.

## Additional file

Additional file 1: Includes: 1.1) Detailed discussion of the Bootstrap approach, 1.2) Detailed discussion of the MVNorm approach, 1.3) Description of the simulation study model, 1.4) Description of the case study model, 1.5) Parameter estimates for the simulation study, 1.6) Kullback-Leibler Divergence for the simulation study, 1.7) Incremental cost-effectiveness planes for all case study analyses, 1.8) CEACs for all case study analyses, and 1.9) References. (PDF 722 kb)

## Abbreviations

Bootstrap: The bootstrap approach
CAPOX-B: Capecitabine, oxaliplatin, and bevacizumab
CEAC: Cost-effectiveness acceptability curve
CR: Complete response
DES: Discrete event simulation
IPD: Individual patient data
MVNorm: The multivariate Normal distributions approach
PFS2: Time until second progression
PR: Partial response
PSA: Probabilistic sensitivity analysis
SD: Stable disease
