# Supplementary material for: Accounting for parameter uncertainty in the definition of parametric distributions used to describe individual patient variation in health economic models
Source: BMC Med Res Methodol. 2017 Dec 15;17:170. doi: 10.1186/s12874-017-0437-y (PMC5732462; doi:10.1186/s12874-017-0437-y)
Supplement: Additional file 1: — Includes: 1.1) Detailed discussion of the Bootstrap approach, 1.2) Detailed discussion of the MVNorm approach, 1.3) Description of the simulation study model, 1.4) Description of the case study model, 1.5) Parameter estimates for the simulation study, 1.6) Kullback-Leibler Divergence for the simulation study, 1.7) Incremental cost-effectiveness planes for all case study analyses, 1.8) CEACs for all case study analyses, and 1.9) References. (PDF 722 kb) [file 12874_2017_437_MOESM1_ESM.pdf]

Additional file 1 to the manuscript:

Accounting for parameter uncertainty in the definition of parametric distributions used to describe individual patient variation in health economic models

by K. Degeling, M.J. IJzerman, M. Koopman, H. Koffijberg

in BMC Medical Research Methodology

Additional file 1 includes:

1.1: Detailed discussion of the Bootstrap approach
1.2: Detailed discussion of the MVNorm approach
1.3: Description of the simulation study model
1.4: Description of the case study model
1.5: Parameter estimates for the simulation study
1.6: Kullback-Leibler Divergence for the simulation study
1.7: Incremental cost-effectiveness planes for all case study analyses
1.8: CEACs for all case study analyses
1.9: References

Additional file 1.1: *Detailed description for the Bootstrap approach*

Non-parametric bootstrapping is a statistical technique that can be used to construct an approximate sampling distribution for a statistic of interest, without the need for assumptions regarding the distribution of this statistic [1]. Several studies investigated the use of bootstrapping in health economics, e.g. for constructing confidence intervals for the incremental cost-effectiveness ratio and the incremental net benefit [2-4]. For a parameter of interest, we are interested in the value for the whole population $\boldsymbol{\beta}$, which cannot be observed. Therefore, we try to find information about the value of this population parameter by drawing a random sample $\mathbf{Y}$ from this population and estimate $\widehat{\boldsymbol{\beta}}(\mathbf{Y})$ the parameter of interest based on this sample. The use of bootstrapping enables us to find information about the relation between the population parameter $\boldsymbol{\beta}$ and its estimate $\widehat{\boldsymbol{\beta}}(\mathbf{Y})$ by the relationship between an observed value for the parameter of interest $\widehat{\boldsymbol{\beta}}(\boldsymbol{y_{obs}})$ and a value for the parameter of interest based on a bootstrap sample $\widehat{\boldsymbol{\beta}}(\mathbf{Y^*})$ [1, 3]. If non-parametric bootstrapping is applied, bootstrap sample $\mathbf{Y^*}$ is constructed by resampling from the observed sample $\mathbf{y_{obs}}$ with replacement [1, 3].

The reasoning for applying non-parametric bootstrapping to reflect the uncertainty in distributions' parameter estimates is explained by the fact that estimates from a clinical trial are almost always obtained based on a part of the total population. Hence, it would be incorrect to assume that the distributions' parameters values estimated from the data are known with certainty, i.e. are correctly describing the entire population. There is a certain sampling error, as another clinical trial may yield different estimates for the distributions' parameters. By bootstrapping trial data, other trial results are simulated and other estimates of parameters will be found, which may generate different probabilistic sensitivity analysis outcomes when Monte Carlo simulation is applied. In fact, as the size $\boldsymbol{n}$ of the sample increases, the estimates obtained by bootstrapping converge to the population value [1, 5].

In the Bootstrap approach the distributions' parameters are repeatedly, say $\boldsymbol{r}$ times, estimated based on different bootstrap samples $\boldsymbol{y_r^*}$ of the original data set $\boldsymbol{y_{obs}}$, resulting in a set of $\{\widehat{\boldsymbol{\beta}}_{1r}^*, \widehat{\boldsymbol{\beta}}_{2r}^*, \ldots, \widehat{\boldsymbol{\beta}}_{ir}^*\}$ parameter bootstrap-estimates, where $\mathbf{i}$ equals the number of parameters required to define the distribution chosen to describe the stochastic uncertainty in the time-to-event data. These bootstrap samples $\boldsymbol{y_r^*}$ are obtained by resampling the original data set $\boldsymbol{y_{obs}}$ with replacement, such that the sizes of the original data set and the bootstrap sample are the same. These $\boldsymbol{r}$ sets of parameter bootstrap estimates $\widehat{\boldsymbol{\beta}}_{ir}^*$ can then be used in the PSA, incorporating one set in each Monte Carlo sample. In short, this approach can be translated into the following four steps:

(1) Generate a feasible bootstrap sample of the original dataset, by resampling this dataset with replacement, such that the sample size of the bootstrap sample equals that of the original dataset.

(2) Fit the pre-specified distribution(s) to the bootstrap sample and record the estimated parameter values, e.g. of the shape and rate parameters of a Gamma distribution.

(3) Repeat (1) and (2) ***r*** times, where ***r*** equals the required number of PSA runs.

(4) Perform the PSA, using a different set of the ***r*** parameter values to define the distribution(s) for each PSA run.

Note that if multiple distributions are fitted in step (2) all of them need to be fitted on the same bootstrap sample to preserve correlation among distributions, which also applies to non-time-to-event distributions, such as Beta distributions to describe utilities, and other model parameters, such as probabilities.

The definition of a feasible bootstrap sample, as required in step (1), may vary between studies and can be difficult to decide on. When repeatedly fitting distributions to these random bootstrap samples, there might be samples for which it is not possible to fit a distribution. For example, a bootstrap sample might be drawn that contains only one time-to-event observation for a specific event, which makes it impossible to fit a Weibull distribution for the time to this event. This particular bootstrap sample can be considered infeasible for fitting a Weibull distribution, though it does contain information on the event of interest and, therefore, might be considered feasible. In such a scenario, it needs to be decided whether the bootstrap sample is to be excluded and a new sample will be drawn. Especially in case of small sample sizes or rare events these situations are likely to occur. Modelers need to be aware of this decision and should clearly communicate their choices in the corresponding publications.

Additional file 1.2: *Detailed description for the MVNorm approach*

The uncertainty in distributions' parameter estimates can be described by assuming these parameter estimates to be Normal distributed. This is appropriate for sufficiently large sample sizes according to the Central Limit Theorem, which states that for any population distribution of a parameter of interest, the distributions of the sample means $\overline{\boldsymbol{\beta}}$ will converge to Normal distributions as the sample size increases [4]. For a sufficient large sample size this indicates that the uncertainty surrounding an independent parameter can be defined according to its estimate $\widehat{\boldsymbol{\beta}}(\boldsymbol{y_{obs}})$ and the Standard Error of the Estimate $\mathbf{SEE}_{\widehat{\boldsymbol{\beta}}(\boldsymbol{y_{obs}})}$:

$$\overline{\boldsymbol{\beta}} \sim \boldsymbol{N}(\widehat{\boldsymbol{\beta}}(\boldsymbol{y_{obs}}), \boldsymbol{SEE}_{\widehat{\boldsymbol{\beta}}(\boldsymbol{y_{obs}})}) \qquad \textit{Equation 1}$$

However, when estimating a multi-parameter distribution, these distribution's parameters are likely to be correlated and it is, therefore, incorrect to define separate Normal distributions using Equation 1 for each of the parameters. However, multivariate Normal distributions can be used to draw correlated values for the parameters of interest. Multivariate Normal distributions are defined by parameter estimates $\{\widehat{\boldsymbol{\beta}}_1, \widehat{\boldsymbol{\beta}}_2, \ldots, \widehat{\boldsymbol{\beta}}_k\}$ and their variance-covariance matrix $\sum_{\boldsymbol{k}}$:

$$\{\overline{\boldsymbol{\beta}}_1, \overline{\boldsymbol{\beta}}_2, \ldots, \overline{\boldsymbol{\beta}}_k\} \sim \boldsymbol{N}_k(\{\widehat{\boldsymbol{\beta}}_1, \widehat{\boldsymbol{\beta}}_2, \ldots, \widehat{\boldsymbol{\beta}}_k\}, \textstyle\sum_{\boldsymbol{k}}) \qquad \textit{Equation 2}$$

Using multivariate Normal distributions, i.e. the MVNorm approach, the uncertainty surrounding the parameter estimates can be approximated using the following four steps:

(1) Fit the pre-specified distribution to the original dataset and record the estimated parameter values, e.g. of the shape and rate parameters of a Gamma distribution, and (calculate) the variance-covariance matrix.

(2) Define a multivariate Normal distribution from the parameters' estimates and their variance-covariance matrix according to (1).

(3) Draw ***r*** feasible sets of parameter values from the defined distribution (2), where ***r*** equals the required number of PSA runs.

(4) Perform the PSA, using a different set of the ***r*** parameter values to define the distribution(s) for each PSA run.

The drawn sets of parameter values need to be assessed for their feasibility, i.e. whether the parameter values are appropriate for the pre-specified distributions. For example, if a Weibull distribution is selected to reflect stochastic uncertainty in time-to-event data, both the distribution's shape and scale parameter values need to be larger than zero, whereas (multivariate) Normal distributions are defined for any real number and may generate negative values. This theoretical definition of feasible parameter values is straightforward, though there might be scenarios in which the drawn parameter values are theoretically feasible, but rather extreme in a practical sense. For example, consider a Weibull distribution with shape and scale parameters of respectively 6 and 150, estimated based on a small sample of time-to-event observations (e.g. n=25), i.e. there might be substantial uncertainty surrounding these estimates. A draw from the corresponding multivariate Normal distribution could return a value for the scale parameter of 300, which is a theoretically feasible value but extreme in a practice sense. This value for the scale parameter will, namely, result in an expected mean value of the corresponding time-to-event distribution that is approximately twice as high with regard to the expected value of the scale parameter (i.e. 150). Although such extreme parameter values may have a substantial impact on modeling outcomes, it is hard to define which values are extreme but rather plausible and which are extreme and implausible.

Additional file 1.3: *Description of the model used in the simulation study*

The model that is used in the simulation study is based on a basic disease progression model including three states: healthy (h), progressed (p), and death (d). The model contains two competing risks for patients in the healthy state, i.e. progression and death. A graphical representation of the disease progression model's structure is presented below:

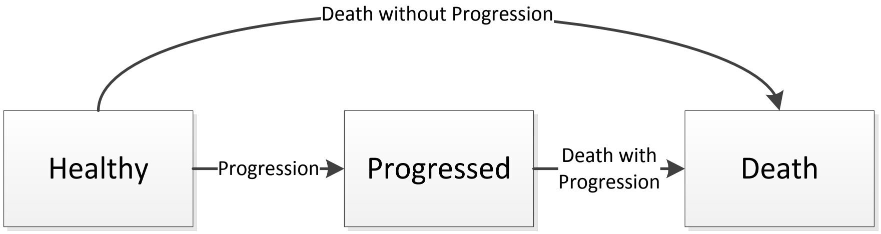

Graphical representation of the model used in the simulation study

Based on this disease progression model, two populations of patients were simulated to sample hypothetical trials from in the simulation study. The parameters defined and the distributions used to do so are defined in the table below. In this table, the control and experimental populations are referred to by a zero (0) and an one (1) in the parameter name, respectively. As can be seen in the table, the two populations differ in terms of treatment costs and survival in the progression state. As explained in the manuscript, the parameter uncertainty surrounding non-time-to-event related variables, i.e. the utilities (u) and costs (c) was reflected according to health economic modeling good practices guidelines. The parameter uncertainty in the defined time-to-event distributions, and their related parameters such as the probability of a competing risks occurring, were addressed according to the Bootstrap or MVNorm approach.

**Model Parameter**	**Population Distribution**	**PSA**	**Description**
Time to Event			
p.hp.0	Probability = 0.6	*According to approach.*	Probability of Progression
t.hp.0	Weibull(shape=1, scale=200)	*According to approach.*	Time to Progression from Healthy
t.hd.0	Weibull(shape=4, scale=150)	*According to approach.*	Time to Death from Health
t.pd.0	Weibull(shape=6, scale=150)	*According to approach.*	Time to Death from Health
p.hp.1	Probability = 0.6	*According to approach.*	Probability of Progression
t.hp.1	Weibull(shape=1, scale=200)	*According to approach.*	Time to Progression from Healthy
t.hd.1	Weibull(shape=4, scale=150)	*According to approach.*	Time to Death from Health
t.pd.1	Weibull(shape=5, scale=350)	*According to approach.*	Time to Death from Health
Effectiveness			
u.healthy	Utility = 0.9	Beta(shape1=9, shape2=1)	Utility in Healthy
u.diseased	Utility = 0.6	Beta(shape1=60, shape2=40)	Utility in Progressed
Costs			
c.progressed.0	Cost = 10	Gamma(shape=10000, rate=1000)	Costs per day
c.progressed.1	Cost = 50	Gamma(shape=500, rate=10)	Costs per day

Additional file 1.4: *Description of the model used in the case study*

The discrete event simulation (DES) model that was used in the case study was defined on patient-level using AnyLogic software and according to the ISPOR-SMDM Modeling Good Research Practice Task Force guidelines [6]. The model was defined to have the same health states as the model that was used for the original evaluation of the CAIRO3 study: post-induction, re-induction, salvage, and death (see figure below) [7].

Weibull distributions [8] were used to define all health state-specific time-to-event parameters and were estimated from the CAIRO3 trial data using the *fitdist* function of the *fitdistrplus* [9] package in R Statistical Software [10]. Events, i.e. transitions between health states, were based on patient-specific processing times, which were randomly drawn from the estimated Weibull distributions. Competing risks were handled by selecting the first event to occur based on the respective observed event probabilities and their corresponding state-specific time-to-event distributions [11]. For example, for a patient that is entering the re-induction state a random number was compared to the chance of progression to determine whether the patient would survive and progress to the salvage therapy state. After the event was selected, the time to that event was randomly drawn from the corresponding Weibull distribution, i.e. time-to-progression or time-to-death.

In the DES model, 10,000 patients were simulated per treatment strategy. Patient-level outcomes were calculated based on the time patients had spent in each health state and were summarized to enable comparison of the two treatment strategies on population level. The DES model was validated according to good practices guidelines by assuring no unnecessary detail was present, structured "walk-throughs", comparing results with calculations by hand, extreme value analysis, trace analysis, sensitivity analysis, and cross validation with the model that was used for the original evaluation of the CAIRO3 study [12, 13].

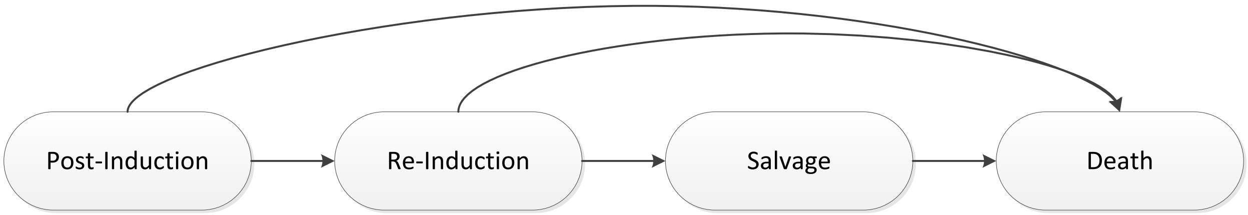

Graphical representation of the model used in the case study

Additional file 1.5: *Mean parameter estimates (including standard error) of the simulation study*

Parameter	Real Value*	n = 500			n = 100			n = 50			n = 25		
		True**	Boot	MVN	True**	Boot	MVN	True**	Boot	MVN	True**	Boot	MVN
p.hp.0	0.60	0.61<br>(0.000)	0.61<br>(0.000)	0.61<br>(0.000)	0.61<br>(0.000)	0.61<br>(0.001)	0.61<br>(0.001)	0.61<br>(0.000)	0.61<br>(0.001)	0.61<br>(0.001)	0.61<br>(0.000)	0.60<br>(0.002)	0.60<br>(0.002)
t.hp.0.shape	1.00	1.00<br>(0.000)	1.00<br>(0.001)	1.00<br>(0.001)	1.01<br>(0.000)	1.04<br>(0.002)	1.02<br>(0.002)	1.04<br>(0.000)	1.09<br>(0.003)	1.04<br>(0.003)	1.09<br>(0.000)	1.22<br>(0.006)	1.10<br>(0.005)
t.hp.0.scale	200.00	199.08<br>(0.000)	199.40<br>(0.237)	199.29<br>(0.237)	198.91<br>(0.000)	201.10<br>(0.559)	200.43<br>(0.558)	199.24<br>(0.000)	202.28<br>(0.788)	200.92<br>(0.786)	199.99<br>(0.000)	205.64<br>(1.155)	203.33<br>(1.152)
t.hd.0.shape	4.00	3.99<br>(0.000)	4.02<br>(0.004)	3.99<br>(0.004)	4.11<br>(0.000)	4.26<br>(0.011)	4.11<br>(0.011)	4.28<br>(0.000)	4.63<br>(0.019)	4.29<br>(0.017)	4.85<br>(0.000)	6.56<br>(0.142)	4.75<br>(0.035)
t.hd.0.scale	150.00	149.14<br>(0.000)	149.02<br>(0.056)	149.06<br>(0.056)	149.05<br>(0.000)	148.67<br>(0.128)	148.89<br>(0.128)	148.73<br>(0.000)	148.14<br>(0.182)	148.61<br>(0.182)	148.13<br>(0.000)	146.86<br>(0.268)	147.88<br>(0.269)
t.pd.0.shape	6.00	6.09<br>(0.000)	6.12<br>(0.006)	6.10<br>(0.006)	6.20<br>(0.000)	6.35<br>(0.013)	6.22<br>(0.013)	6.36<br>(0.000)	6.65<br>(0.020)	6.37<br>(0.020)	6.74<br>(0.000)	7.45<br>(0.038)	6.72<br>(0.032)
t.pd.0.scale	150.00	149.54<br>(0.000)	149.51<br>(0.029)	149.51<br>(0.029)	149.42<br>(0.000)	149.35<br>(0.066)	149.40<br>(0.066)	149.28<br>(0.000)	149.03<br>(0.094)	149.16<br>(0.094)	149.12<br>(0.000)	148.29<br>(0.139)	148.63<br>(0.139)
p.hp.1	0.60	0.61<br>(0.000)	0.61<br>(0.000)	0.61<br>(0.000)	0.61<br>(0.000)	0.61<br>(0.001)	0.61<br>(0.001)	0.61<br>(0.000)	0.61<br>(0.001)	0.61<br>(0.001)	0.61<br>(0.000)	0.60<br>(0.002)	0.60<br>(0.002)
t.hp.1.shape	1.00	1.02<br>(0.000)	1.02<br>(0.001)	1.02<br>(0.001)	1.04<br>(0.000)	1.06<br>(0.002)	1.04<br>(0.002)	1.06<br>(0.000)	1.11<br>(0.003)	1.06<br>(0.003)	1.12<br>(0.000)	1.24<br>(0.006)	1.12<br>(0.005)
t.hp.1.scale	200.00	197.24<br>(0.000)	197.44<br>(0.231)	197.37<br>(0.231)	198.06<br>(0.000)	198.07<br>(0.523)	197.68<br>(0.524)	198.91<br>(0.000)	199.70<br>(0.728)	198.86<br>(0.728)	198.94<br>(0.000)	201.34<br>(1.068)	199.87<br>(1.068)
t.hd.1.shape	4.00	3.97<br>(0.000)	4.00<br>(0.004)	3.97<br>(0.004)	4.08<br>(0.000)	4.22<br>(0.011)	4.08<br>(0.011)	4.25<br>(0.000)	4.59<br>(0.018)	4.25<br>(0.016)	4.70<br>(0.000)	8.11<br>(1.828)	4.69<br>(0.033)
t.hd.1.scale	150.00	149.47<br>(0.000)	149.49<br>(0.056)	149.52<br>(0.056)	149.21<br>(0.000)	149.00<br>(0.125)	149.21<br>(0.125)	149.17<br>(0.000)	148.64<br>(0.187)	149.11<br>(0.187)	148.36<br>(0.000)	147.56<br>(0.276)	148.57<br>(0.277)
t.pd.1.shape	5.00	4.96<br>(0.000)	4.97<br>(0.004)	4.95<br>(0.004)	5.07<br>(0.000)	5.15<br>(0.011)	5.04<br>(0.010)	5.20<br>(0.000)	5.41<br>(0.017)	5.17<br>(0.016)	5.43<br>(0.000)	6.02<br>(0.029)	5.43<br>(0.025)
t.pd.1.scale	350.00	349.95<br>(0.000)	349.84<br>(0.085)	349.90<br>(0.085)	349.57<br>(0.000)	349.69<br>(0.190)	349.99<br>(0.190)	349.53<br>(0.000)	348.39<br>(0.274)	349.00<br>(0.274)	348.79<br>(0.000)	346.40<br>(0.403)	347.67<br>(0.403)
incr.costs		9444.90<br>(0.000)	8921.80<br>(7.357)	8914.76<br>(7.350)	9421.60<br>(0.000)	8928.78<br>(16.884)	8909.90<br>(16.832)	9419.53<br>(0.000)	8923.27<br>(24.560)	8898.46<br>(24.415)	9448.78<br>(0.000)	8852.58<br>(33.291)	*unrealistic*<br>*unrealistic*
incr.effects		0.18<br>(0.000)	0.18<br>(0.001)	0.18<br>(0.001)	0.18<br>(0.000)	0.18<br>(0.001)	0.18<br>(0.001)	0.18<br>(0.000)	0.18<br>(0.002)	*unrealistic*<br>*unrealistic*	0.18<br>(0.000)	0.17<br>(0.003)	*unrealistic*<br>*unrealistic*

* The real value refers to the value that was used to define the simulated populations (Online Resource 3). ** The true value refers to the "true" value as defined in the methods section in the manuscript, i.e. the value representing the scenario in which 2500 clinical studies were performed to estimate the parameter.

Additional file 1.6: *Mean Kullback-Leibler Divergence results (including standard error) for the simulation study*

	n = 500		n = 100		n = 50		n = 25	
	Boot	MVN	Boot	MVN	Boot	MVN	Boot	MVN
p.hp.0	0.46 (0.013)	0.48 (0.014)	0.49 (0.014)	0.50 (0.014)	0.48 (0.014)	0.50 (0.014)	0.50 (0.015)	0.52 (0.015)
t.hp.0.shape	0.50 (0.015)	0.49 (0.014)	0.59 (0.017)	0.55 (0.016)	0.70 (0.021)	0.61 (0.016)	0.84 (0.026)	0.67 (0.017)
t.hp.0.scale	0.45 (0.012)	0.46 (0.012)	0.48 (0.012)	0.50 (0.012)	0.48 (0.012)	0.50 (0.012)	0.50 (0.012)	0.52 (0.013)
t.hd.0.shape	0.50 (0.015)	0.48 (0.014)	0.60 (0.017)	0.55 (0.015)	0.72 (0.020)	0.62 (0.015)	0.60 (0.020)	0.50 (0.011)
t.hd.0.scale	0.48 (0.013)	0.48 (0.013)	0.52 (0.013)	0.52 (0.013)	0.56 (0.014)	0.56 (0.014)	0.68 (0.017)	0.68 (0.017)
t.pd.0.shape	0.51 (0.015)	0.48 (0.013)	0.58 (0.017)	0.51 (0.014)	0.66 (0.018)	0.56 (0.014)	0.75 (0.021)	0.61 (0.014)
t.pd.0.scale	0.46 (0.013)	0.47 (0.013)	0.49 (0.013)	0.49 (0.013)	0.52 (0.014)	0.51 (0.013)	0.59 (0.015)	0.58 (0.015)
p.hp.1	0.48 (0.013)	0.46 (0.013)	0.52 (0.015)	0.50 (0.014)	0.54 (0.016)	0.52 (0.015)	0.52 (0.016)	0.50 (0.014)
t.hp.1.shape	0.50 (0.014)	0.49 (0.014)	0.58 (0.017)	0.55 (0.015)	0.66 (0.020)	0.59 (0.015)	0.82 (0.025)	0.65 (0.016)
t.hp.1.scale	0.45 (0.012)	0.47 (0.012)	0.46 (0.012)	0.48 (0.012)	0.44 (0.011)	0.46 (0.012)	0.47 (0.012)	0.50 (0.012)
t.hd.1.shape	0.49 (0.014)	0.47 (0.013)	0.61 (0.018)	0.51 (0.014)	0.69 (0.020)	0.55 (0.014)	0.84 (0.024)	0.64 (0.015)
t.hd.1.scale	0.46 (0.013)	0.46 (0.012)	0.48 (0.012)	0.48 (0.012)	0.56 (0.014)	0.57 (0.015)	0.69 (0.017)	0.70 (0.017)
t.pd.1.shape	0.49 (0.015)	0.48 (0.014)	0.56 (0.016)	0.53 (0.014)	0.65 (0.019)	0.57 (0.014)	0.77 (0.023)	0.61 (0.015)
t.pd.1.scale	0.48 (0.013)	0.48 (0.012)	0.48 (0.012)	0.48 (0.012)	0.52 (0.013)	0.52 (0.013)	0.57 (0.014)	0.57 (0.014)
incr.costs	0.39 (0.006)	0.39 (0.006)	0.14 (0.003)	0.14 (0.003)	0.09 (0.002)	0.09 (0.002)	0.05 (0.001)	0.05 (0.001)
incr.effects	0.39 (0.011)	0.39 (0.011)	0.51 (0.014)	0.49 (0.014)	0.53 (0.015)	0.48 (0.013)	0.05 (0.005)	0.05 (0.004)
average	0.47	0.46	0.50	0.49	0.55	0.51	0.58	0.52

Additional file 1.7: *Incremental cost-effectiveness planes for the cohort and all subgroups of the case study*

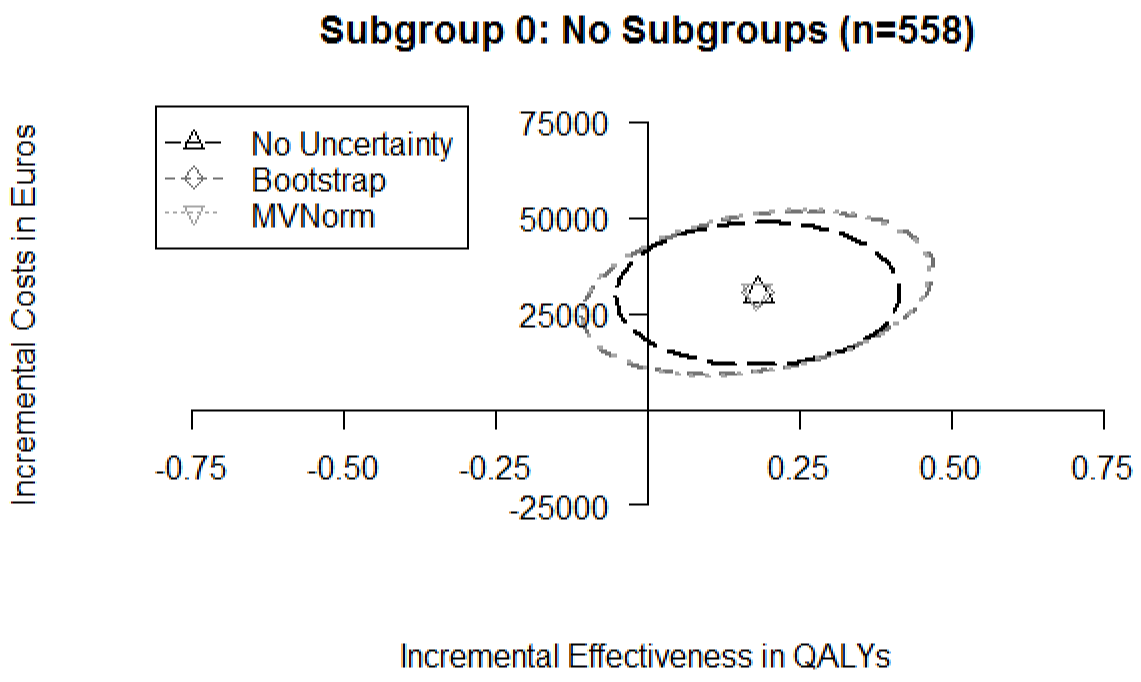

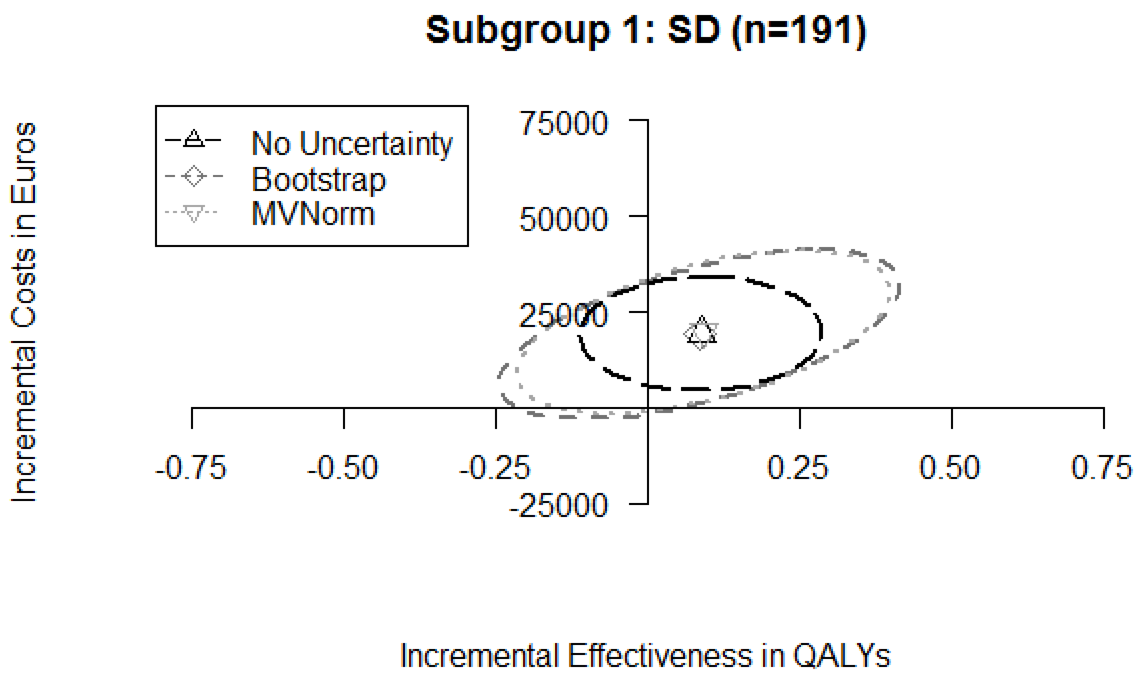

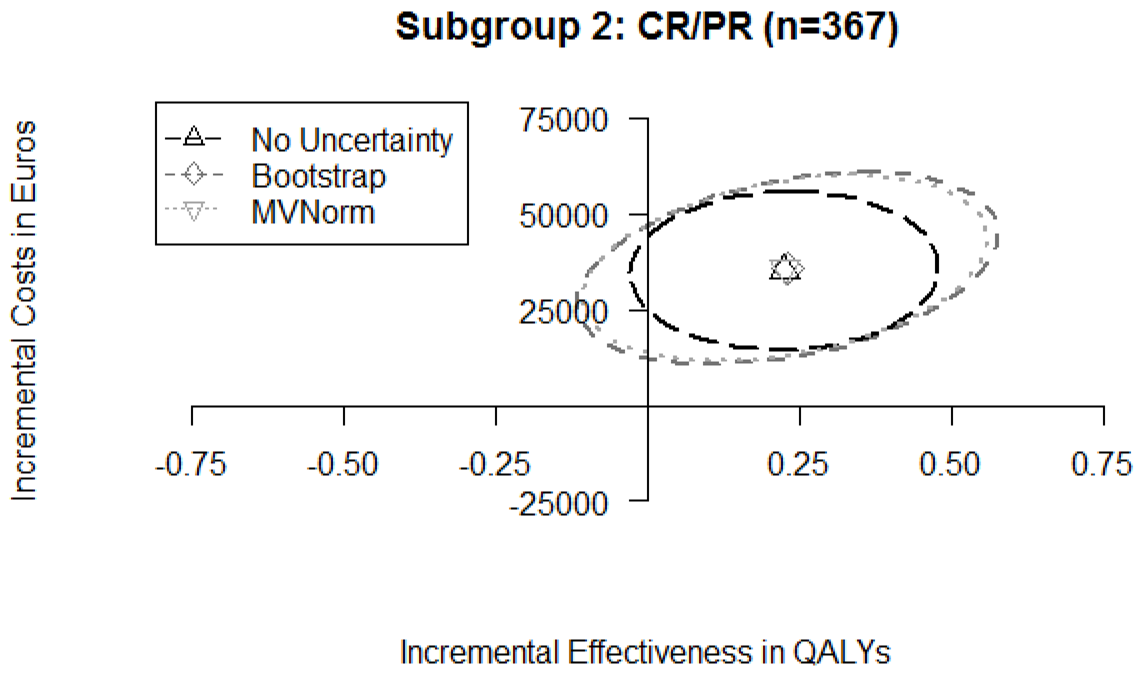
Subgroup 2: CR/PR (n=367)
No Uncertainty
Bootstrap
MVNorm
75000
50000
25000
-25000
-0.75
-0.50
-0.25
0.25
0.50
0.75
Incremental Costs in Euros
Incremental Effectiveness in QALYs

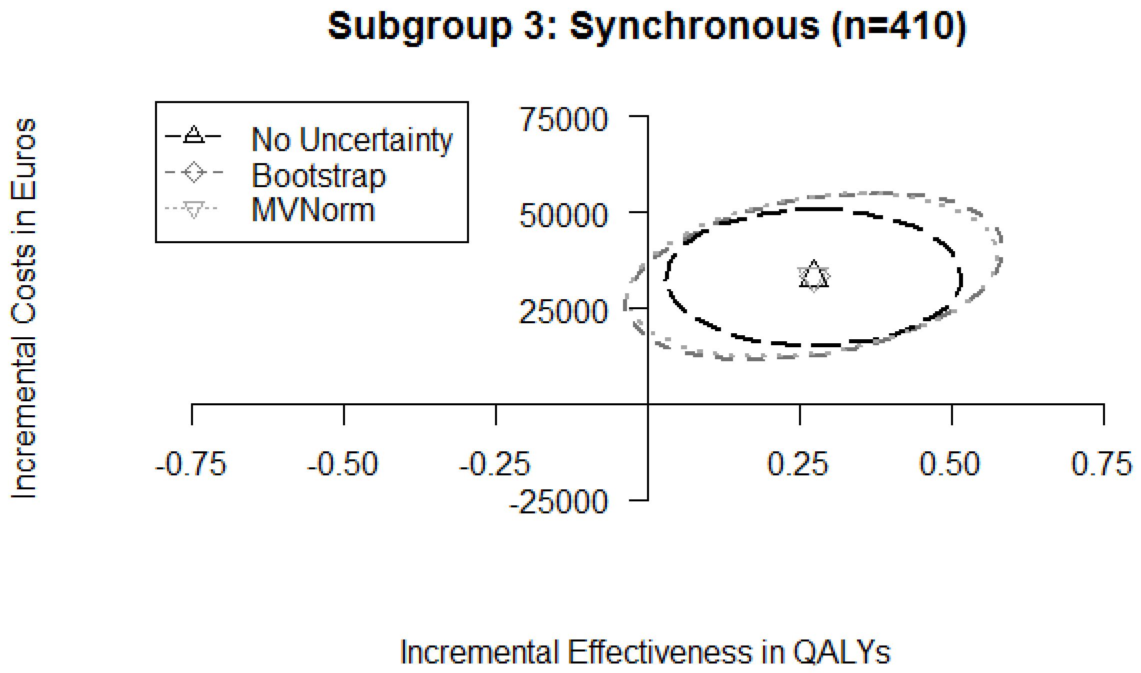
Subgroup 3: Synchronous (n=410)
No Uncertainty
Bootstrap
MVNorm
75000
50000
25000
-25000
-0.75
-0.50
-0.25
0.25
0.50
0.75
Incremental Costs in Euros
Incremental Effectiveness in QALYs

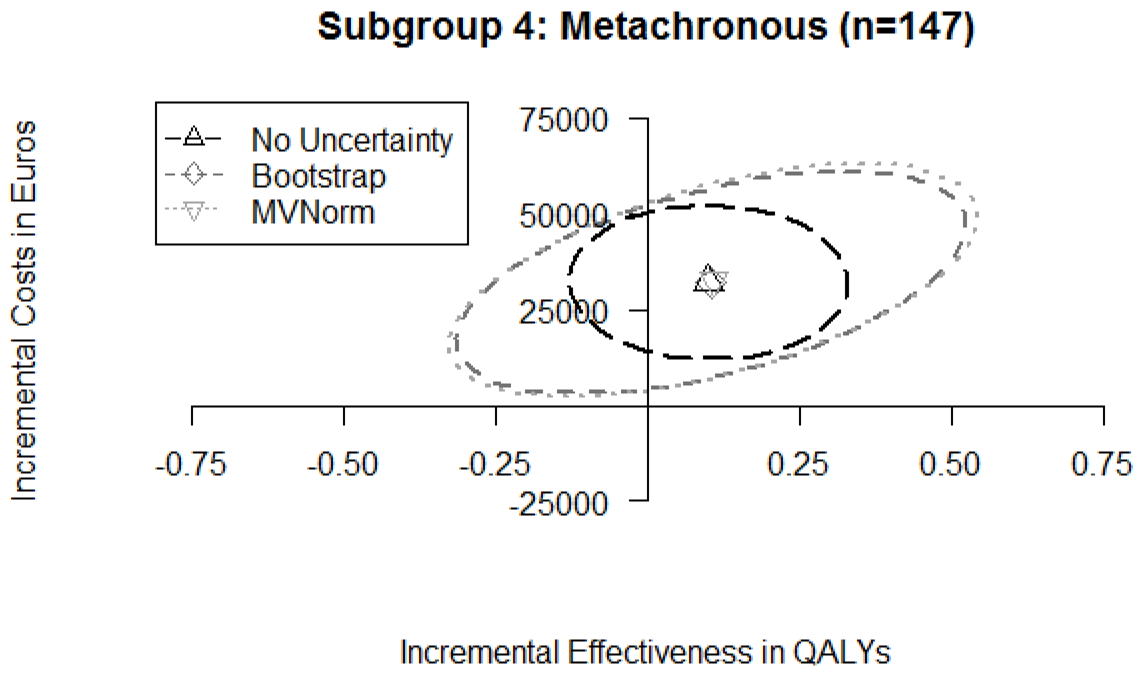
Subgroup 4: Metachronous (n=147)
No Uncertainty
Bootstrap
MVNorm
75000
50000
25000
-25000
-0.75
-0.50
-0.25
0.25
0.50
0.75
Incremental Costs in Euros
Incremental Effectiveness in QALYs

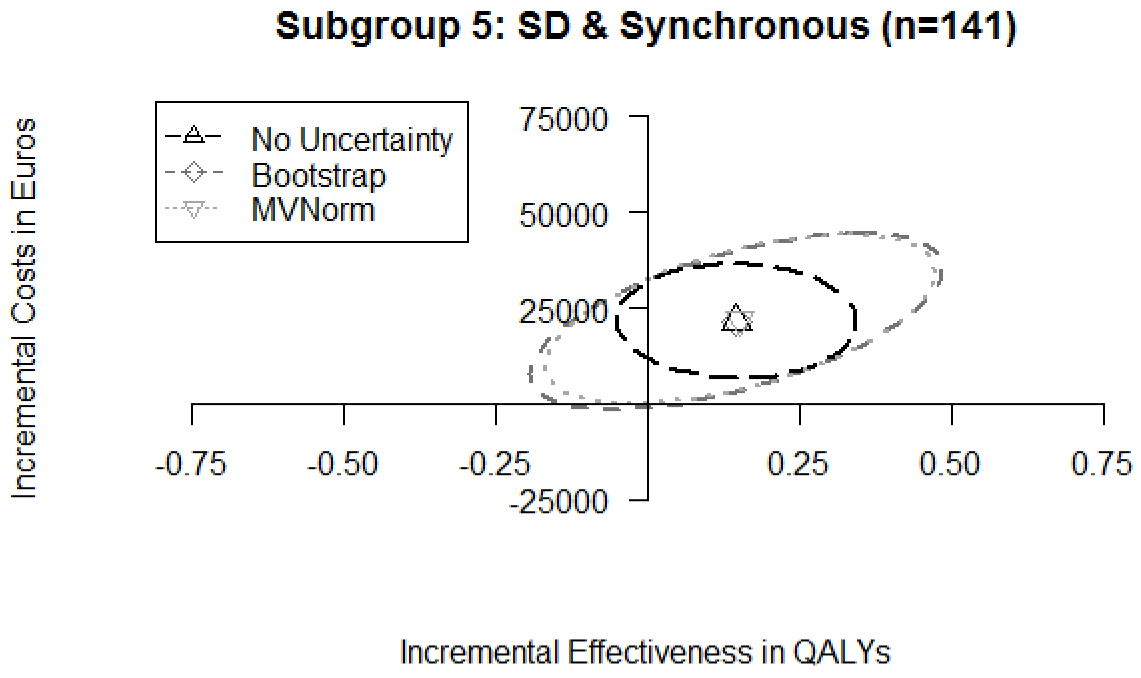
Subgroup 5: SD & Synchronous (n=141)
No Uncertainty
Bootstrap
MVNorm
75000
50000
25000
-25000
-0.75
-0.50
-0.25
0.25
0.50
0.75
Incremental Costs in Euros
Incremental Effectiveness in QALYs

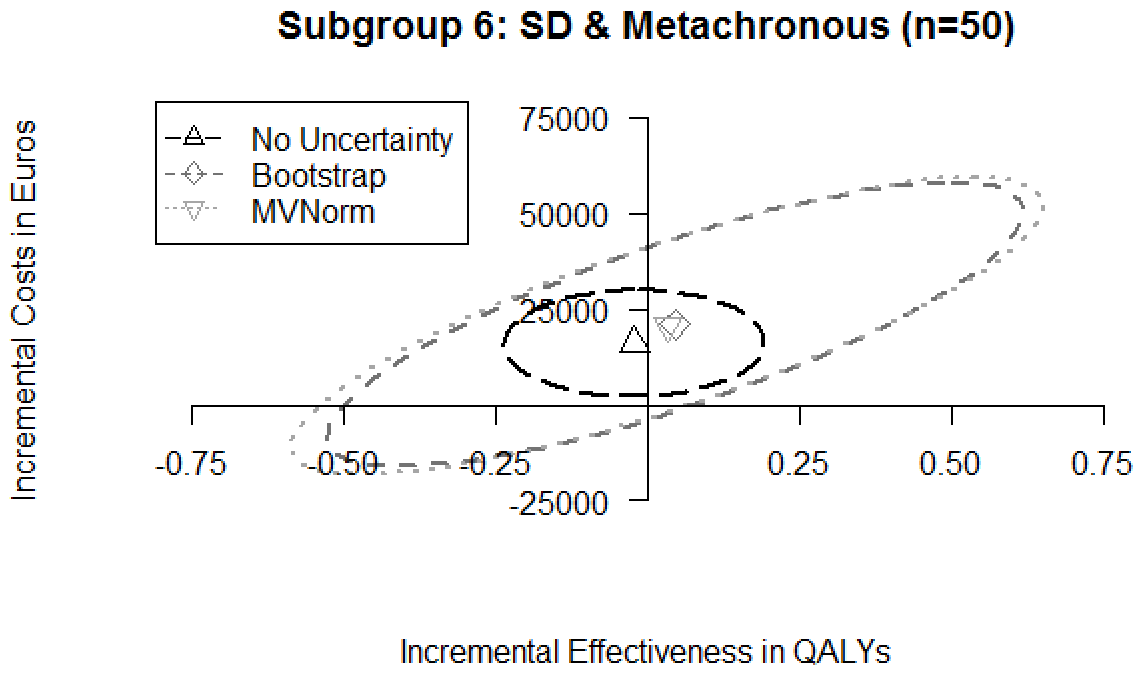
Subgroup 6: SD & Metachronous (n=50)
No Uncertainty
Bootstrap
MVNorm
75000
50000
25000
-25000
-0.75
-0.50
-0.25
0.25
0.50
0.75
Incremental Costs in Euros
Incremental Effectiveness in QALYs

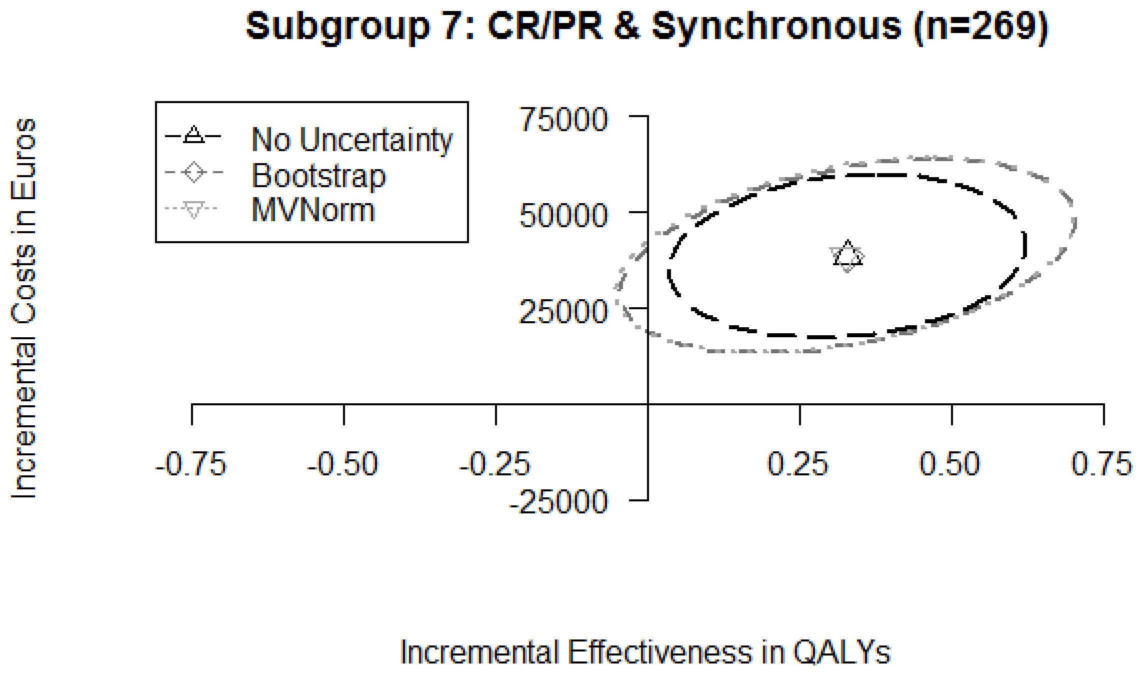
Subgroup 7: CR/PR & Synchronous (n=269)
No Uncertainty
Bootstrap
MVNorm
75000
50000
25000
-25000
-0.75
-0.50
-0.25
0.25
0.50
0.75
Incremental Costs in Euros
Incremental Effectiveness in QALYs

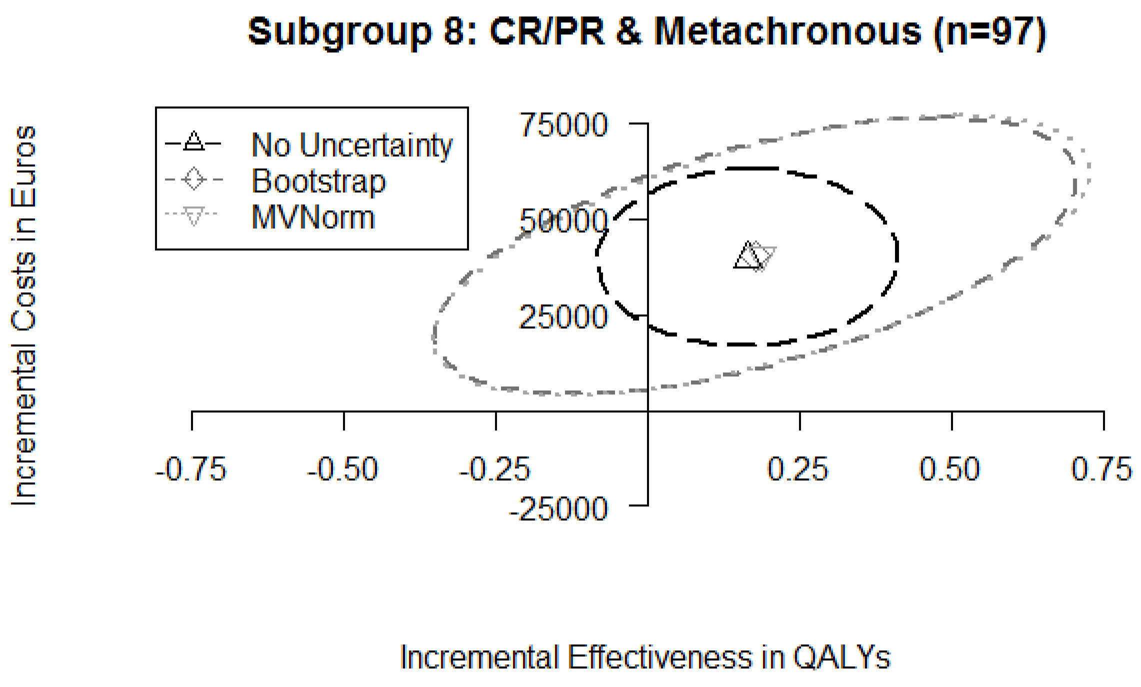
Subgroup 8: CR/PR & Metachronous (n=97)
No Uncertainty
Bootstrap
MVNorm
75000
50000
25000
-25000
-0.75
-0.50
-0.25
0.25
0.50
0.75
Incremental Costs in Euros
Incremental Effectiveness in QALYs

Additional file 1.8: *Cost-Effectiveness Acceptability Curves for the cohort and all subgroups of the case study*

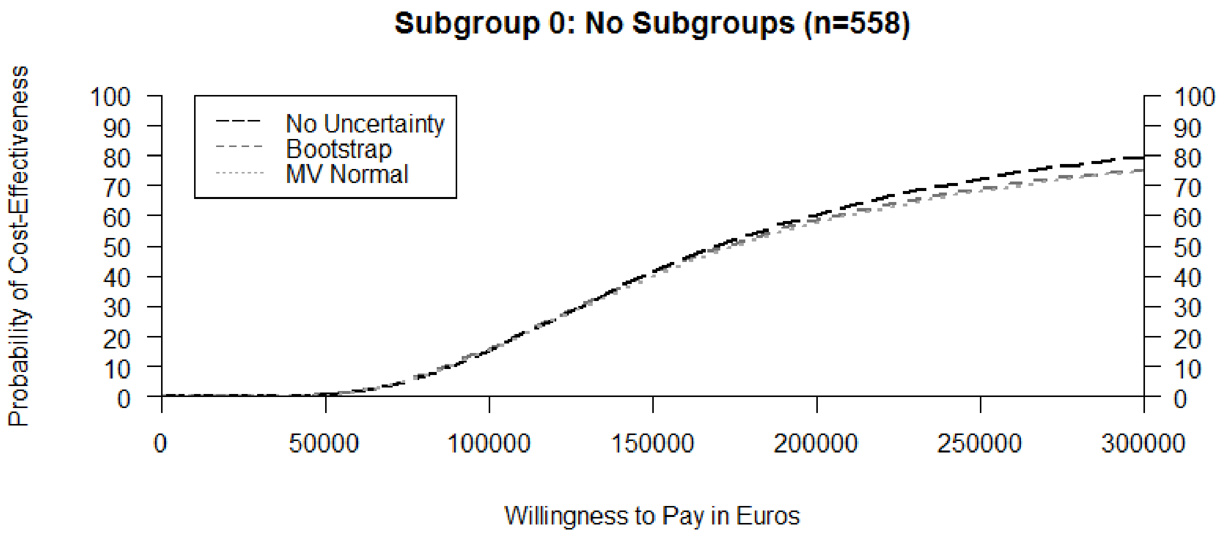

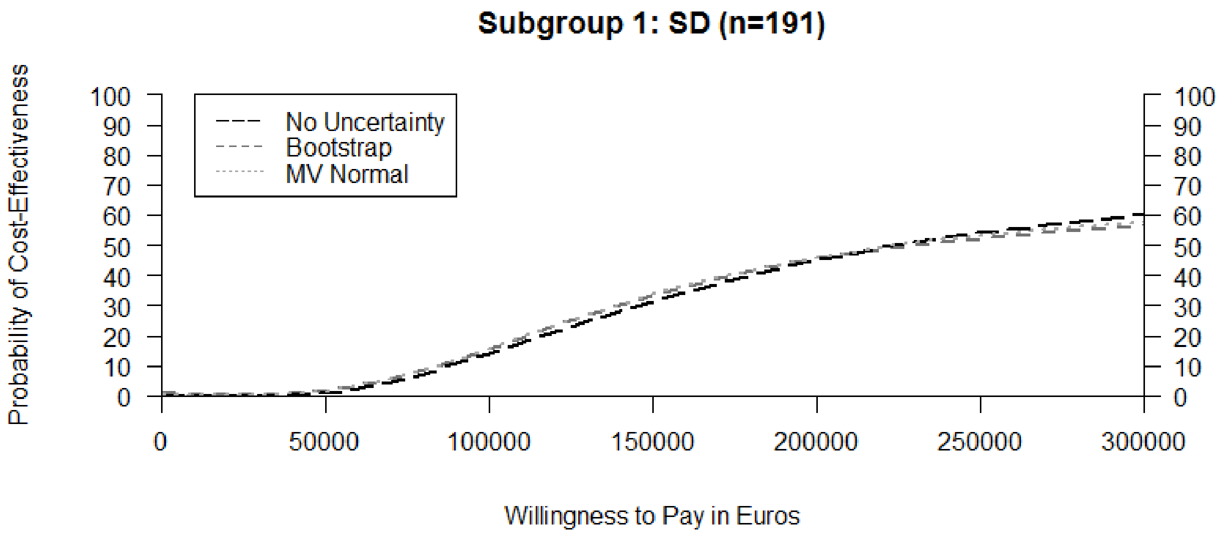

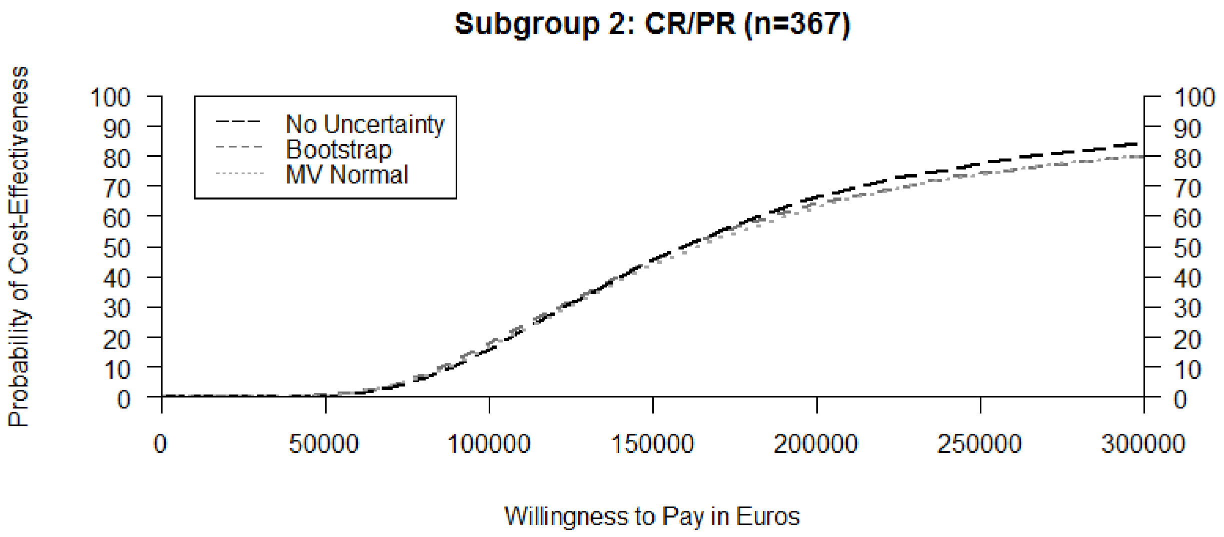
Subgroup 2: CR/PR (n=367)
Probability of Cost-Effectiveness
No Uncertainty
Bootstrap
MV Normal
0
10
20
30
40
50
60
70
80
90
100
0
50000
100000
150000
200000
250000
300000
Willingness to Pay in Euros

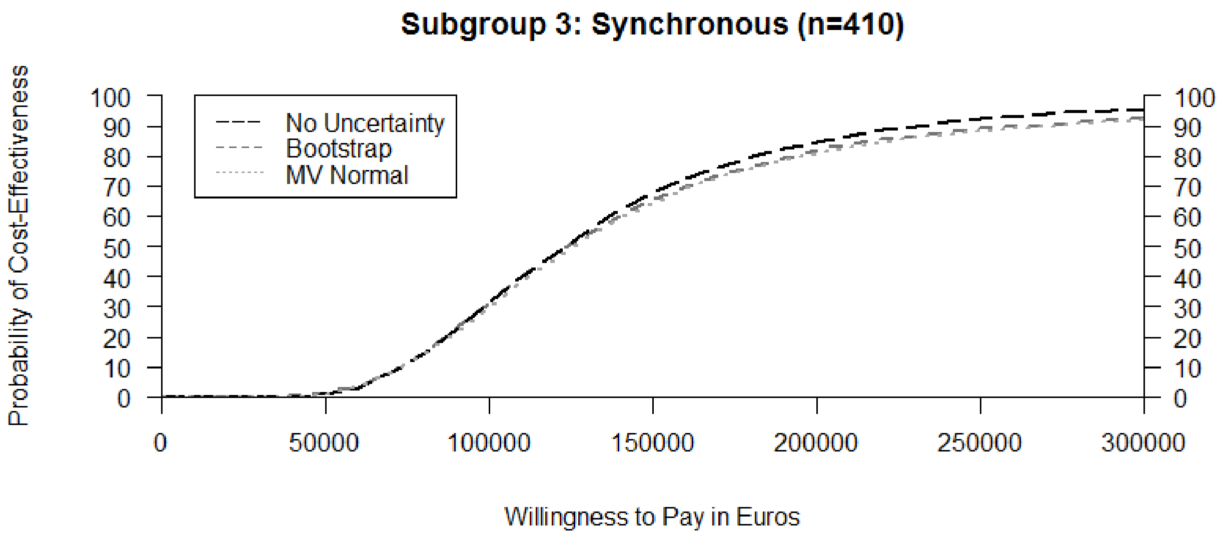
Subgroup 3: Synchronous (n=410)
Probability of Cost-Effectiveness
No Uncertainty
Bootstrap
MV Normal
0
10
20
30
40
50
60
70
80
90
100
0
50000
100000
150000
200000
250000
300000
Willingness to Pay in Euros

Subgroup 4: Metachronous (n=147)

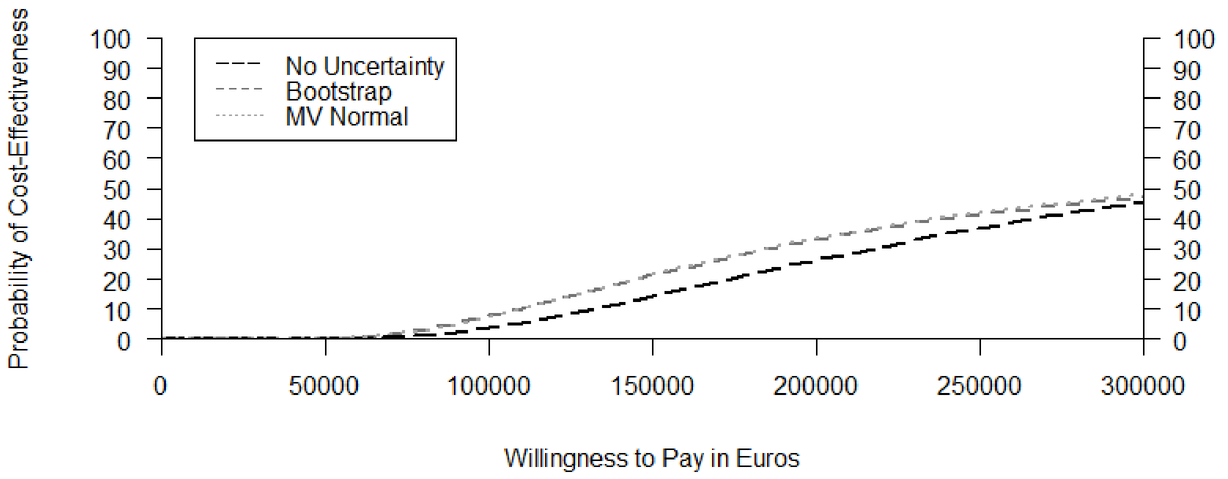

Subgroup 5: SD & Synchronous (n=141)

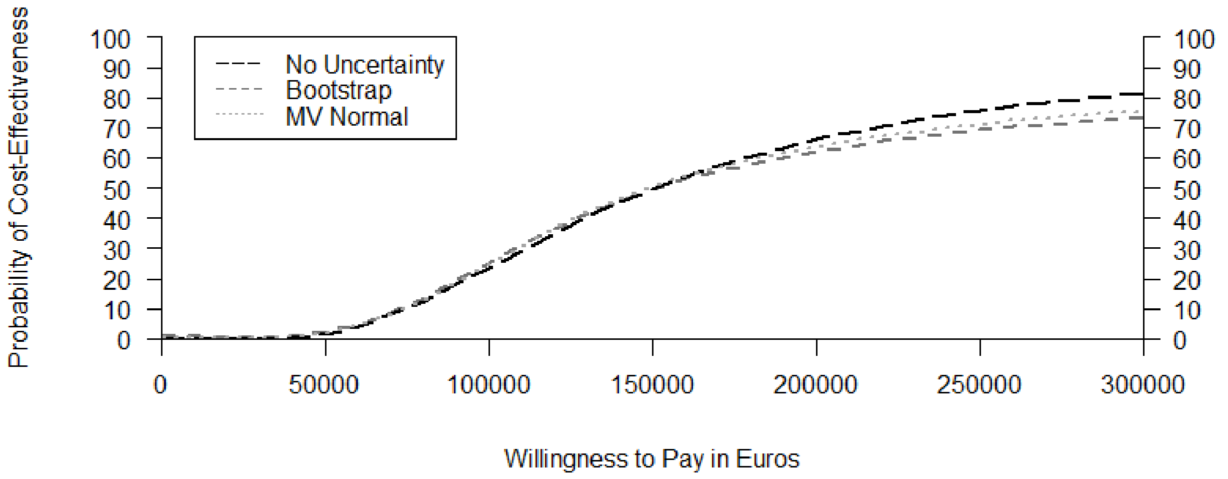

Subgroup 6: SD & Metachronous (n=50)

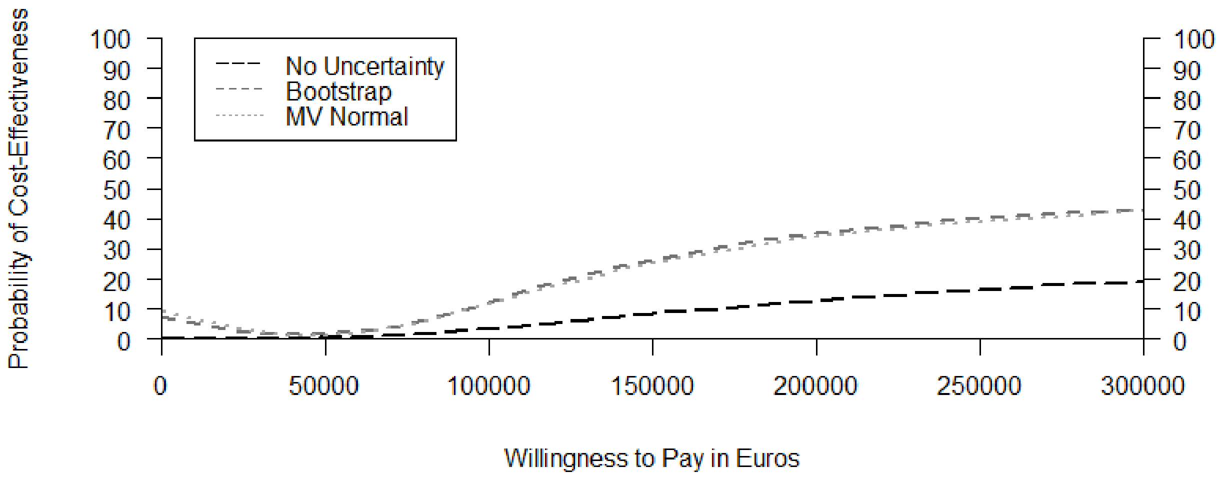

Subgroup 7: CR/PR & Synchronous (n=269)

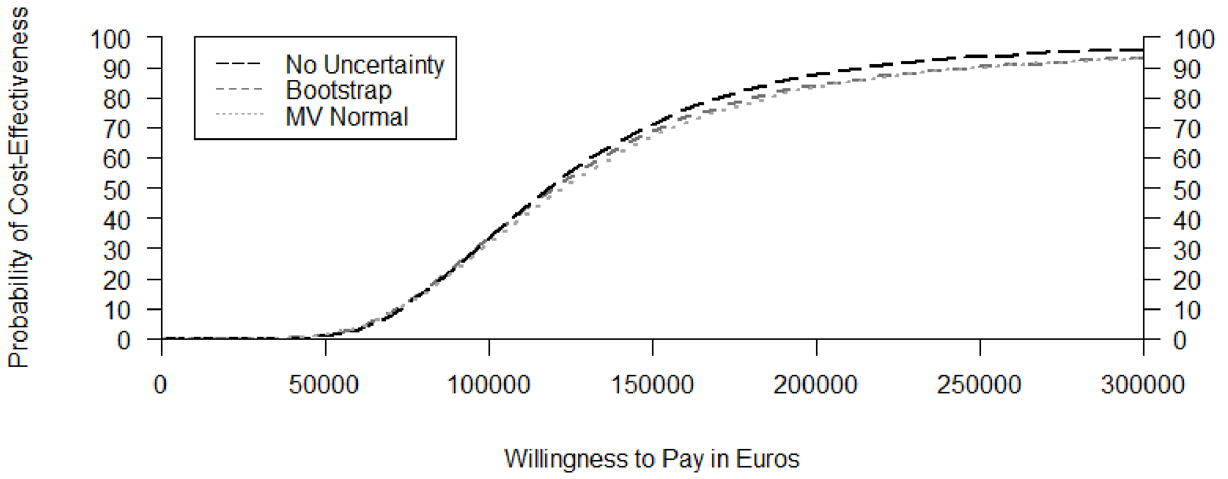

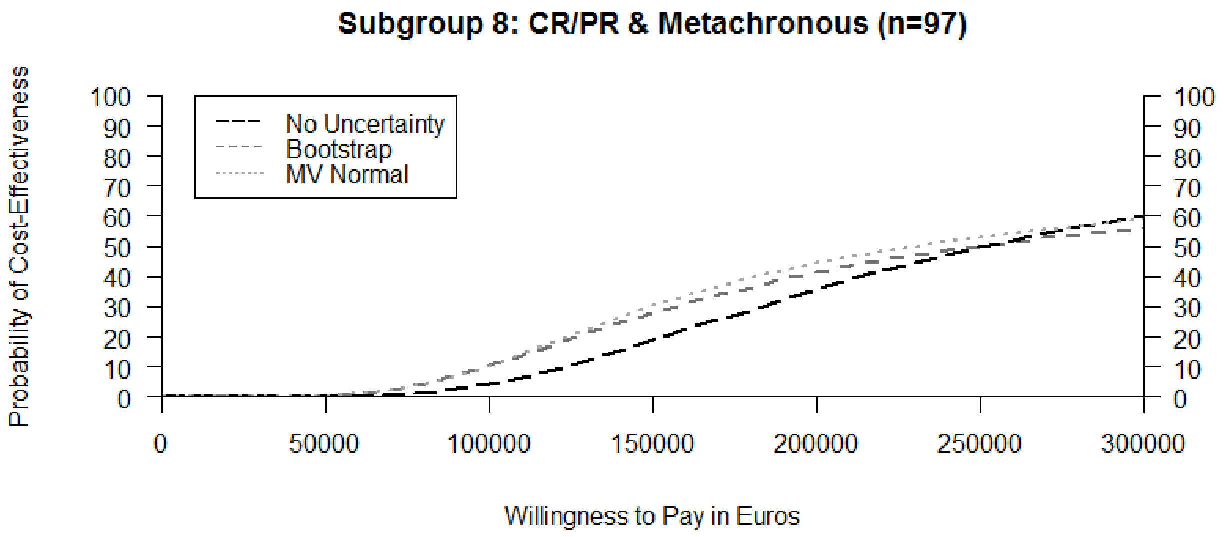
Subgroup 8: CR/PR & Metachronous (n=97)
Probability of Cost-Effectiveness
No Uncertainty
Bootstrap
MV Normal
0
10
20
30
40
50
60
70
80
90
100
0
50000
100000
150000
200000
250000
300000
Willingness to Pay in Euros

Additional file 1.9: *References*

1. Davison AC, Hinkley. DV. Bootstrap Methods and their Application. 1st ed. Cambridge University Press; 1997.

2. Briggs AH, Wonderling DE, Mooney CZ. Pulling cost-effectiveness analysis up by its bootstraps: a non-parametric approach to confidence interval estimation. Health economics. 1997;6(4):327-40.

3. Carpenter J, Bithell J. Bootstrap confidence intervals: when, which, what? A practical guide for medical statisticians. Stat Med. 2000;19(9):1141-64.

4. Nixon RM, Wonderling D, Grieve RD. Non-parametric methods for cost-effectiveness analysis: the central limit theorem and the bootstrap compared. Health Economics. 2010;19(3):316-33.

5. Efron B, Tibshirani RJ. An introduction to the bootstrap. CRC press; 1994.

6. Karnon J, Stahl J, Brennan A, Caro JJ, Mar J, Möller J. Modeling using Discrete Event Simulation: A Report of the ISPOR-SMDM Modeling Good Research Practices Task Force-4. Value in Health. 2012;15(6):821-7.

7. Franken MD, van Rooijen EM, May AM, Koffijberg H, van Tinteren H, Mol L, ten Tije AJ, Creemers GJ, van der Velden AMT, Tanis BC, Uyl-de Groot CA, Punt CJA, Koopman M, van Oijen MGH. Cost-effectiveness of capecitabine and bevacizumab maintenance treatment after first-line induction treatment in metastatic colorectal cancer. European Journal of Cancer. 2017;75:204-12.

8. Collett D. Modelling Survival Data in Medical Research, Second Edition. Taylor & Francis; 2003.

9. Delignette-Muller ML, Dutang C. fitdistrplus: An R Package for Fitting Distributions. Journal of Statistical Software. 2015;64(4):1-34.

10. R Core Team (2016). R: A language and environment for statistical computing. R Foundation for Statistical Computing, Vienna, Austria. URL https://www.R-project.org/.

11. Barton P, Jobanputra P, Wilson J, Bryan S, Burls A. The use of modelling to evaluate new drugs for patients with a chronic condition: the case of antibodies against tumour necrosis factor in rheumatoid arthritis. Health Technology Assessment. 2004;8(11):104.

12. Eddy DM, Hollingworth W, Caro JJ, Tsevat J, McDonald KM, Wong JB. Model Transparency and Validation: A Report of the ISPOR-SMDM Modeling Good Research Practices Task Force-7. Value in Health. 2012;15(6):843-50.

13. Vemer P, Corro Ramos I, van Voorn GAK, Al MJ, Feenstra TL. AdViSHE: A Validation-Assessment Tool of Health-Economic Models for Decision Makers and Model Users. PharmacoEconomics. 2016;34:349-61.
